# Efficient calculation of carrier scattering rates from first principles

**DOI:** 10.1038/s41467-021-22440-5

**Published:** 2021-04-13

**Authors:** Alex M. Ganose, Junsoo Park, Alireza Faghaninia, Rachel Woods-Robinson, Kristin A. Persson, Anubhav Jain

**Affiliations:** 1 Energy Technologies Area, Lawrence Berkeley National Laboratory, Berkeley, CA USA; 2 Applied Science and Technology Graduate Group, University of California, Berkeley, CA USA; 3 Department of Materials Science and Engineering, University of California, Berkeley, CA USA; 4 Molecular Foundry, Energy Sciences Area, Lawrence Berkeley National Laboratory, Berkeley, CA USA

**Keywords:** Thermoelectrics, Computational methods, Electronic properties and materials

## Abstract

The electronic transport behaviour of materials determines their suitability for technological applications. We develop a computationally efficient method for calculating carrier scattering rates of solid-state semiconductors and insulators from first principles inputs. The present method extends existing polar and non-polar electron-phonon coupling, ionized impurity, and piezoelectric scattering mechanisms formulated for isotropic band structures to support highly anisotropic materials. We test the formalism by calculating the electronic transport properties of 23 semiconductors, including the large 48 atom CH_3_NH_3_PbI_3_ hybrid perovskite, and comparing the results against experimental measurements and more detailed scattering simulations. The Spearman rank coefficient of mobility against experiment (*r*_s_ = 0.93) improves significantly on results obtained using a constant relaxation time approximation (*r*_s_ = 0.52). We find our approach offers similar accuracy to state-of-the art methods at approximately 1/500th the computational cost, thus enabling its use in high-throughput computational workflows for the accurate screening of carrier mobilities, lifetimes, and thermoelectric power.

## Abstract (web summary)

It is difficult to compute the transport properties of a broad array of complex materials both accurately and inexpensively. Here, the authors develop a computationally efficient method for calculating carrier scattering rates of semiconductors, with good accuracy but low cost.

## Introduction

Solid-state materials exhibit a variety of electronic transport behaviors, enabling their deployment in a variety of technological applications, including light-emitting devices, photocatalysts, transparent conductors, solar cells, and thermoelectrics^1–6^. Recent years have seen an explosion of interest into the computational prediction of electronic transport properties, leading to a hierarchy of methods that can be broadly split into three categories. (i) Semi-empirical models for approximating electron lifetimes have been employed since the 1930s^7–12^ but have seen a resurgence with the advent of large-scale materials science databases due to their computational efficiency^13–15^. These approaches have recently been extended to permit first-principles inputs^16–19^ but the underlying assumption of single parabolic bands with no anisotropy limits their widespread application^20^. (ii) The second category eschews the calculation of electron lifetimes, instead of employing a constant scattering rate for all electronic states. When combined with Fourier^21,22^ or Wannier^23^ interpolation of ab initio electronic band structures this enables efficient calculation of transport properties in complex systems with multiple non-parabolic bands^24–26^. Recent work has applied this approach to compute the transport behavior of large numbers of materials, including 48,000 semiconductors in the Materials Project database by Ricci et al.^27^, 809 sulfides by Miyata et al.^28^, and 75 potential thermoelectric candidates by Xing et al.^29^; however, the unphysical treatment of electron scattering and the reliance on an empirical tuning parameter often results in significant errors. (iii) Finally, the fully first principles approach to calculating the electron–phonon interaction based on density functional perturbation theory (DFPT) combined with Wannier interpolation can now yield highly accurate electron lifetimes and have demonstrated remarkable agreement to experimental measurements of electron mobility and conductivity^30–35^. The calculation of the scattering matrix elements needed to obtain electron lifetimes is highly computationally demanding, even when approximations are made. With few exceptions^36–38^, such approaches have been applied to highly symmetric systems with limited numbers of atoms.^39–44^. Although the computational cost of mobility calculations can be reduced though energy-averaging of the matrix elements^45^, the initial DFPT calculation needed to obtain the matrix elements typically represents the majority of the computational expense. Despite the range of computational techniques available, no existing method can be applied to compute the transport properties of a broad array of complex materials both accurately and inexpensively. This limitation is a primary obstacle in the application of high-throughput computations to the search for novel functional materials as well as applying this theory to larger and more complex materials.

In the present work, we develop an efficient formalism for calculating anisotropic transport properties of semiconductors that is accurate over a range of materials and amenable to use in high-throughput computational workflows. Our approach relies on inputs that can be obtained from low-cost ab initio methods and that are routinely available in computational materials science databases. Scattering rates are calculated using the momentum relaxation time approximation (MRTA) to the Boltzmann transport equation (BTE). The present method includes fully anisotropic acoustic deformation potential, piezoelectric, ionized impurity, and polar electron–phonon scattering. As an initial test of the approach, we calculate the temperature-dependent electron mobility and Seebeck coefficient of 23 semiconductors including the large 48-atom CH_3_NH_3_PbI_3_ hybrid perovskite. The Spearman rank coefficient of mobility against experiment (*r*_s_ = 0.93) improves significantly on results obtained using a constant relaxation time approximation (*r*_s_ = 0.52). Furthermore, we find our approach offers similar accuracy to state-of-the art methods at 1/500th the computational cost. An open source software implementation of the method is made freely available.

## Results

### Computationally efficient matrix elements

The scattering rate of an electron from an initial state *n***k**, where *n* is a band index and **k** is a wave vector, to final state *m***k** + **q** is described by Fermi’s golden rule as

1 $${\tau }_{n{\bf{k}}\to m{\bf{k}}+{\bf{q}}}^{-1}=\frac{2\pi }{\hslash }| {g}_{nm}({\bf{k}},{\bf{q}}){| }^{2}\delta \left({\varepsilon }_{n{\bf{k}}}-{\varepsilon }_{m{\bf{k}}+{\bf{q}}}\right),$$

where *ℏ* is the reduced Planck’s constant, *ε* is the electron energy, *δ* is the Dirac delta function and *g* is the coupling matrix element. The above equation is given for the case of perfectly elastic scattering^46^, in which electrons do not gain or lose energy during the scattering process. A similar equation can be defined for inelastic processes (Supplementary Eq. 7), for instance to describe scattering that occurs via emission or absorption of a phonon. In general, however, the impact of different scattering mechanisms is expressed via the coupling matrix element ${g}_{nm}({\bf{k}},{\bf{q}})=\langle m{\bf{k}}+{\bf{q}}|{{{\Delta }}}_{{\bf{q}}}V|n{\bf{k}}\rangle$ where Δ_**q**_*V* is an electronic perturbation of some kind. The primary obstacle in obtaining accurate transport properties is evaluating *g*_*nm*_(**k**, **q**) on dense Brillouin zone grids, which has so far proven computationally prohibitive for all but the simplest systems^47,48^.

Historically, this challenge has been avoided. In the constant relaxation time approximation (CRTA), Eq. (1) is simplified to a single constant. An alternative is to employ model matrix elements formulated for isotropic band structures based on intrinsic materials parameters. For example, the treatment of deformation potential scattering due to long-wavelength acoustic phonons proposed by Bardeen and Shockley^8^ depends only on an averaged elastic constant and band edge deformation potential; it ignores perturbations from transverse phonon modes and anisotropy in the deformation response. This simple approach has been employed widely in computations of acoustic phonon scattering but is unreliable and does not generalise to complex systems or metals^49–51^. An alternative approach, developed by Khan and Allen^49^, can reproduce the fully first principles electron–phonon scattering rate if the strain tensor caused by the phonon and an additional velocity term are included. The resulting matrix element is given by

2 $${g}_{nm}^{{\rm{KA}}}=\left\langle m{\bf{k}}+{\bf{q}}\right|{{\bf{S}}}_{{\bf{q}}}:({{\bf{D}}}_{n{\bf{k}}}+{{\bf{v}}}_{n{\bf{k}}}\otimes {{\bf{v}}}_{n{\bf{k}}})|n{\bf{k}}\rangle,$$

where : denotes the double dot product, **S**_**q**_ is the strain associated with an acoustic phonon, **D**_*n***k**_ is the second rank deformation potential tensor and **v**_*n***k**_ is the group velocity. The velocity term is essential to correct the deformation potential in metals and at states away from the valence or conduction band edge in semiconductors. In practice, however, this equation is no longer simple to evaluate as it requires knowledge of the atomic displacements (the polarization direction) of the phonon mode in order to obtain the strain tensor.

In the present work, we combine the simplicity of the Bardeen-Shockley approach with the accuracy of the Khan-Allen matrix element by exploiting the acoustoelastic properties of materials. The dispersion relations for acoustic waves are contained in the Christoffel equation^52^

3 $$\left[{{{\Gamma }}}_{\hat{{\bf{q}}}}-\rho {c}^{2}{\mathbb{1}}\right]\hat{{\bf{u}}}=0,$$

where 1 is the identity matrix, $\hat{{\bf{q}}}$ and $\hat{{\bf{u}}}$ are unit vectors giving the direction of phonon propagation and polarization, respectively, *ρ* is the density, *c* is the wave velocity, and ${{{\Gamma }}}_{\hat{{\bf{q}}}}={\bf{C}}\hat{{\bf{q}}}\cdot \hat{{\bf{q}}}$ is the Christoffel matrix where **C** is the rank 4 elastic constant tensor. Solving the Christoffel equation for a phonon wave vector direction ($\hat{{\bf{q}}}$) results in three sets of eigenvalues (*ρc*^2^) and eigenvectors ($\hat{{\bf{u}}}$), that correspond to the (quasi-)longitudinal and (quasi-)transverse normal modes of the material. The unit strain associated with each mode is given by $\hat{{\bf{S}}}=\hat{{\bf{q}}}\otimes \hat{{\bf{u}}}$ and the amplitude of the strain at any temperature *T* can be obtained from the potential energy of the acoustic phonon as $\sqrt{{k}_{{\rm{B}}}T/\rho {c}^{2}}$, where *k*_B_ is the Boltzmann constant^53^. From this we arrive at an expression for acoustic deformation potential scattering (ad) that relies only on the deformation potentials and elastic constants and includes scattering from longitudinal and transverse modes in a single matrix element, given in the Born approximation^54^ as

4 $${g}_{nm}^{{\rm{ad}}}({\bf{k}},{\bf{q}})=\sqrt{{k}_{{\rm{B}}}T}\mathop{\sum}\limits _{{\bf{G}}\ne -{\bf{q}}}\left[\frac{{\tilde{{\bf{D}}}}_{n{\bf{k}}}:{\hat{{\bf{S}}}}_{l}}{{c}_{l}\sqrt{\rho }}+\frac{{\tilde{{\bf{D}}}}_{n{\bf{k}}}:{\hat{{\bf{S}}}}_{{t}_{1}}}{{c}_{{t}_{1}}\sqrt{\rho }}+\frac{{\tilde{{\bf{D}}}}_{n{\bf{k}}}:{\hat{{\bf{S}}}}_{{t}_{2}}}{{c}_{{t}_{2}}\sqrt{\rho }}\right]\left\langle m{\bf{k}}+{\bf{q}}\right|{e}^{i({\bf{q}}+{\bf{G}})\cdot {\bf{r}}}|n{\bf{k}}\rangle$$

where ${\tilde{{\bf{D}}}}_{n{\bf{k}}}={{\bf{D}}}_{n{\bf{k}}}+{{\bf{v}}}_{n{\bf{k}}}\otimes {{\bf{v}}}_{n{\bf{k}}}$, and the subscripts *l*, *t*_1_, and *t*_2_ indicate properties belonging to the longitudinal and transverse modes.

Scattering by acoustic phonons through the piezoelectric interaction (pi) occurs in non-centrosymmetric systems and can dominate at low temperatures (≲50 K). We have applied a similar treatment to extend the isotropic matrix element of Meijer and Polder^11^, Harrison^55^, and Zook^53^, to include the full piezoelectric stress tensor **h** and scattering from all three acoustic modes. The resulting matrix element is given by

5 $${g}_{nm}^{{\rm{pi}}}({\bf{k}},{\bf{q}})=\sqrt{{k}_{{\rm{B}}}T}\mathop{\sum} _{{\bf{G}}\ne -{\bf{q}}}\left[\frac{\hat{{\bf{n}}}{\bf{h}}:{\hat{{\bf{S}}}}_{l}}{{c}_{l}\sqrt{\rho }}+\frac{\hat{{\bf{n}}}{\bf{h}}:{\hat{{\bf{S}}}}_{{t}_{1}}}{{c}_{{t}_{1}}\sqrt{\rho }}+\frac{\hat{{\bf{n}}}{\bf{h}}:{\hat{{\bf{S}}}}_{{t}_{2}}}{{c}_{{t}_{2}}\sqrt{\rho }}\right]\frac{\left\langle m{\bf{k}}+{\bf{q}}\right|{e}^{i({\bf{q}}+{\bf{G}})\cdot {\bf{r}}}|n{\bf{k}}\rangle}{\left|{\bf{q}}+{\bf{G}}\right|},$$

where $\hat{{\bf{n}}}=({\bf{q}}+{\bf{G}})/\left|{\bf{q}}+{\bf{G}}\right|$ is a unit vector in the direction of scattering. Due to the small energies of long-wavelength acoustic phonons, both piezoelectric and acoustic deformation potential scattering describe a purely elastic process.

We treat polar optical phonon scattering (po) by extending the Frölich model^12^ to include quantum mechanical wave function overlaps and anisotropic permittivity. Here, electrons in a dielectric medium are perturbed by a dispersionless longitudinal optical phonon mode with frequency *ω*_po_. Our electron–phonon matrix element takes the form

6 $${g}_{nm}^{{\rm{po}}}({\bf{k}},{\bf{q}})	= {\left[\frac{\hslash {\omega }_{{\rm{po}}}}{2}\right]}^{1/2}\mathop{\sum} _{{\bf{G}}\ne -{\bf{q}}}{\left(\frac{1}{\hat{{\bf{n}}}\cdot {{\boldsymbol{\epsilon }}}_{\infty }\cdot \hat{{\bf{n}}}}-\frac{1}{\hat{{\bf{n}}}\cdot {{\boldsymbol{\epsilon }}}_{{\rm{s}}}\cdot \hat{{\bf{n}}}}\right)}^{1/2}\\ 	\quad\times \frac{\left\langle m{\bf{k}}+{\bf{q}}\right|{e}^{i({\bf{q}}+{\bf{G}})\cdot {\bf{r}}}|n{\bf{k}}\rangle}{\left|{\bf{q}}+{\bf{G}}\right|},$$

where ***ϵ***_s_ and ***ϵ***_*∞*_ are the static and high-frequency dielectric tensors. To capture scattering from the full phonon band structure in a single phonon frequency, each phonon mode is weighted by the dipole moment it produces (Supplementary Eq. 29) in line with recent work that has rederived the Frölich model for systems with multiple phonon branches^56,57^. Both our extension of the Frölich model and state-of-the-art first principles approaches produce similar matrix elements in the long-wavelength limit that dominates scattering (due to the polar singularity at **q** → 0^56^).

Following the classic treatment of Brooks and Herring^9,58^ we consider the scattering from fully ionized impurities (ii) modeled as screened Coulomb potentials, with the matrix element given by

7 $${g}_{nm}^{{\rm{ii}}}({\bf{k}},{\bf{q}})=\mathop{\sum} _{{\bf{G}}\ne -{\bf{q}}}\frac{{n}_{{\rm{ii}}}^{1/2}Ze}{\hat{{\bf{n}}}\cdot {{\boldsymbol{\epsilon }}}_{{\rm{s}}}\cdot \hat{{\bf{n}}}}\frac{\left\langle m{\bf{k}}+{\bf{q}}\right|{e}^{i({\bf{q}}+{\bf{G}})\cdot {\bf{r}}}|n{\bf{k}}\rangle}{{\left|{\bf{q}}+{\bf{G}}\right|}^{2}+{\beta }^{2}},$$

where *Z* is the charge state of the impurity center, *e* is the electron charge, *n*_ii_ = (*n*_h_ − *n*_e_)/*Z* is the concentration of ionized impurities, and *β* is the inverse screening length (Supplementary Eq. 10). Unlike previous formulations, our matrix element accounts for anisotropy in the charge screening through use of the full dielectric tensor. Taken together, Eqs. (1) and (7) reveal that, assuming weak screening, the scattering almost diverges at long wavelengths (**q** → 0) due to a $1/{\left|{\bf{q}}\right|}^{4}$ dependence, and therefore requires very fine sampling to describe correctly. For this reason, almost all attempts at calculating electron scattering by ionized impurities have employed the Brooks–Herring formula, in which Eq. (7) is analytically integrated over the phase space for an isotropic parabolic band^32,59^. To overcome this limitation, we employ a modified linear-tetrahedron approach to integration, in which tetrahedron cross sections are numerically resampled with hundreds of extra points that exactly satisfy the delta term in Eq. (1). This allows for effective **k**-point mesh densities that would be almost impossible to achieve with uniform **k**-point sampling (the full methodology is provided in the Supplementary Methods). While other works have used similar matrix elements^60^, we extend these approaches by considering interband scattering and first-principles wave function overlaps in the evaluation of Coulomb-based impurity scattering. In Supplementary Fig. 5, we demonstrate that our methodology reproduces the exact Brooks–Herring mobility for parabolic band structures and reveal the failure of the Brooks–Herring approach in the case of systems containing multiple anisotropic valleys.

The final **k**-dependent scattering rates are obtained by integrating Eq. (1) over all phonon wave vectors (**q**) in the first Brillouin zone. Elastic scattering processes are well described by the MRTA to the BTE due to the requirement that *τ*_*n***k**→*m***k**+**q**_ = *τ*_*m***k**+**q**→*n***k**_^40^. As this condition does not hold for inelastic processes, we adopt the self-energy relaxation time approximation (SERTA) to obtain the final polar phonon coupling rates^32^. Further justification for this approach is detailed in the Supplementary Methods. Electronic eigenvalues and group velocities needed to calculate scattering and transport properties are Fourier interpolated onto dense Brillouin zone grids using the BoltzTraP2 software^22^ (as detailed in the Supplementary Methods). Electron mobility and Seebeck coefficient are calculated using the linearized BTE via the Onsager transport coefficients^22,61^ (Supplementary Eqs. 13 to 15). We also employ a custom procedure for selecting the most important **k**-points at which to calculate scattering to further reduce the computational expense (detailed in the Supplementary Methods).

Unlike other state-of-the-art approaches in which a computationally expensive DFPT calculation is required to obtain *g*(**k**, **q**), in our method all matrix elements depend only on common materials parameters (*ω*_po_, *ϵ*_s_, *ϵ*_∞_, etc.) that can be calculated relatively inexpensively. Crucially, many of these properties are already tabulated in databases such as the Materials Project^62^ or can be obtained through relatively cheap ab initio calculations. Furthermore, the matrix elements can be evaluated in a fixed time regardless of the number of atoms in the system, and multiple temperatures and carrier concentrations can be calculated simultaneously with only a modest increase in the computational time. Full-timing information for the calculation of all first-principles inputs required to compute the transport properties of the materials discussed in this work and the scaling performance of each code routine is given in the Supplementary Methods.

### Analysis of scattering rates and electron mobility

**Fig. 1:**
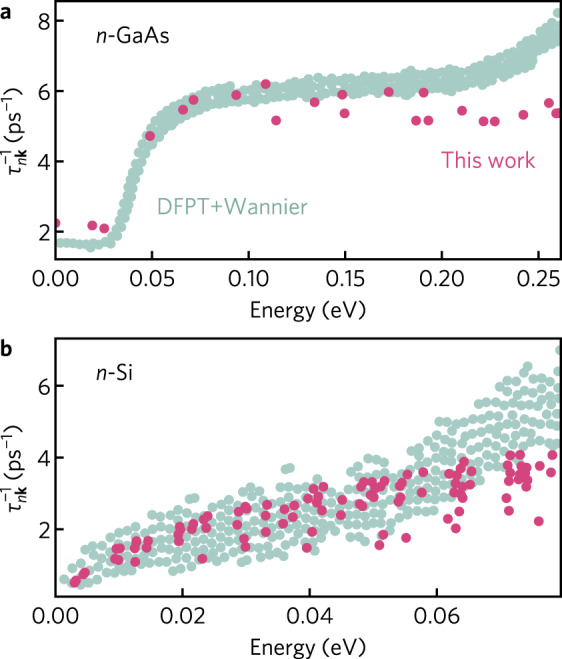
Calculated scattering rates. Comparison of the calculated (pink) scattering rates, ${\tau }_{n{\bf{k}}}^{-1}$, against those obtained using density functional perturbation theory combined with Wannier interpolation (DFPT + Wannier, light teal) for (**a**) *n*-GaAs^44^ and (**b**) *n*-Si^32^ at 300 K.

In Fig. 1, we compare mode-dependent scattering rates for *n*-Si and *n*-GaAs calculated by our method against fully first principles calculations (DFPT + Wannier) at 300 K obtained using the EPW and PERTURBO softwares^32,35^. The scattering of electrons in Si is dominated by acoustic phonons whereas polar optical phonon scattering dominates in GaAs, as revealed by the mobility analysis in Supplementary Fig. 9. Excellent agreement is seen for both systems, with the onset of polar optical emission scattering in GaAs well described by our calculations. The high degree of agreement for Si is somewhat surprising considering our calculations do not include optical phonons that are known to contribute to scattering at room temperature. However, as we do not consider symmetry selection rules in our calculations, the acoustic phonon scattering rate will be slightly overestimated and is expected to partially pick up some of the missing optical deformation scatterings. Whereas in Si, group theoretical rules dictate that intervalley *g*-phonon transitions are only possible through longitudinal optical phonons, Rode^63^ and others^64^ have demonstrated the temperature-dependent transport behavior can reproduced considering only acoustic phonons if selection processes are ignored. Additional comparisons against DFPT + Wannier scattering rates for 3C-SiC and *p*-SnSe are provided in Supplementary Fig. 13. In both cases, the shape and magnitude of the scattering rates is well reproduced, particularly at low energies, despite the simpler approach that does not involve an expensive DFPT calculation to obtain the matrix elements.

**Fig. 2:**
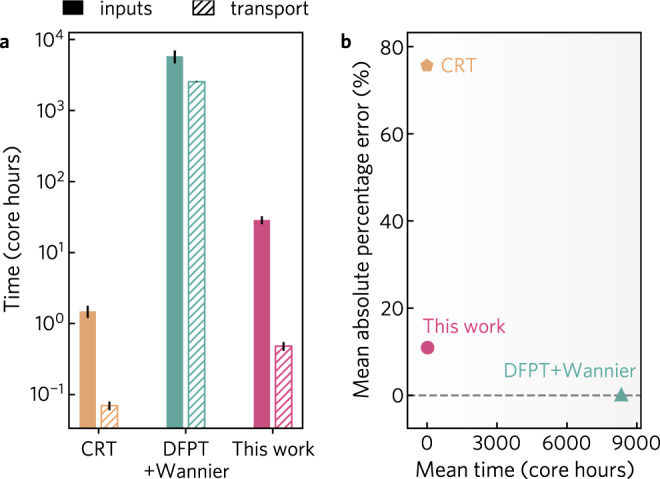
Comparison of speed against accuracy for transport calculations. Existing methods for calculating electron transport properties are either computationally efficient but inaccurate (constant relaxation time, CRT, orange) or accurate but highly computationally demanding (density functional perturbation theory combined with Wannier interpolation, DFPT + Wannier, teal). The approach outlined in this work (pink) demonstrates accuracy comparable to state-of-the-art methods at ~1/500th of the computational cost. **a** The time required to obtain electron mobility for each method is broken down by the time spent computing first-principles inputs and performing the scattering and transport calculations. **b** The mean absolute percentage error in the calculated mobility at 300 K is compared to the total computational time (including the time to obtain all first-principles inputs). Results are averaged for NbFeSb (*p*-type, *n* = 2 × 10^20^ cm^−3^, DFPT + Wannier^39,90^) and Ba_2_BiAu (*n*-type, *n* = 1 × 10^14^ cm^−3^, DFPT + Wannier^91^). In (**b**), the mobility error is referenced with respect to state-of-the-art DFPT + Wannier calculations as high-quality experimental data were not available. The full timing breakdown for each material is provided in Supplementary Tables 2 and 3. Constant relaxation time calculations were performed with *τ* = 10 fs.

In Fig. 2, we compare the time taken to compute the transport properties of NbFeSb and Ba_2_BiAu (the full-timing breakdown is tabulated in Supplementary Tables 2 and 3). Taking into account the time required to compute all first-principles inputs and the electron mobility at a single temperature and carrier concentration, our method offers over a 2 order of magnitude speed up compared to DFPT + Wannier (an average of 29 core hours versus 8350 core hours). Considering only the time needed to obtain the scattering rates and transport properties (i.e., presuming all inputs have already been tabulated), our approach offers a 4 order of magnitude speed up (Fig. 2a). This can be exploited when performing calculations at multiple temperatures and carrier concentrations. For example, calculating the mobility of Ba_2_BiAu for 10 temperatures requires ~32,000 core hours using DFPT+Wannier compared to <35 core hours with our approach (95% of which is required to calculate the first principles inputs). Furthermore, we expect the relative cost advantage of our method to increase with system size as unlike in DFPT + Wannier the computational expense of the matrix elements does not depend on the number of atoms. This reduction in computational time, combined with similar accuracy to DFPT + Wannier [within 10%, see Fig. 2b], makes our approach amenable to the large-scale calculation of electronic transport properties.

**Fig. 3:**
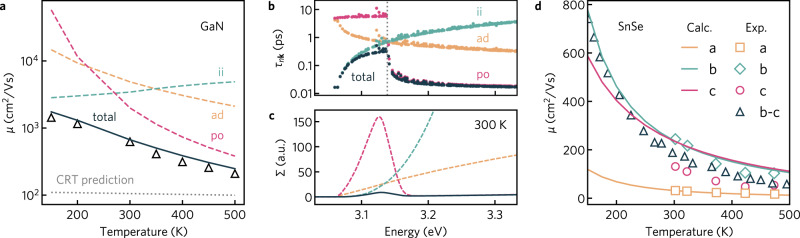
Temperature-dependent transport properties of GaN and SnSe. **a** Comparison of the electron mobility, *μ*, of GaN against experiment (black triangles,^92^). Mobility limited by ionized impurity (teal, ii), acoustic deformation potential (orange, ad), and polar optical phonon scattering (pink, po) is indicated in dashed lines. Total mobility taking into account all scattering mechanisms ($1/{\tau }_{n{\bf{k}}}^{{\rm{ii}}}+1/{\tau }_{n{\bf{k}}}^{{\rm{ad}}}+1/{\tau }_{n{\bf{k}}}^{{\rm{po}}}$) is given by the black solid line. Constant relaxation time (CRT) calculations with *τ* = 10 fs is given by dotted gray line. **b** Electron lifetimes, *τ*_*n***k**_ and (**c**) spectral conductivity, Σ, arising from different scattering processes in GaN at 300 K. The valence band maximum is set to zero eV. In (**b**), the vertical dotted gray line indicates the energy of the effective polar phonon frequency, *ω*_po_. **d** Comparison of the direction-dependent mobility of SnSe against experiments—*a* (orange), *b* (teal), *c* (pink) points from ref. ^66^, *b*–*c* (black) points from ref. ^93^.

Figure 3a plots the calculated mobility of GaN against experimental measurements, indicating very close agreement from 150 to 500 K. As each scattering mechanism is treated with a separate matrix element, this allows the impact of individual scattering processes to be assessed. At low temperatures, the mobility of GaN is limited by impurity scattering, with polar optical phonon scattering dominating above 300 K, as illustrated by the dashed lines in Fig. 3a. The total mobility taking into account all scattering mechanisms reproduces the experimental mobility with very high agreement. Further insight into the competing nature of the scattering mechanisms is provided by the energy dependence of the electron lifetimes and the resulting spectral conductivity, Σ(*ε*) = *v*(*ε*)^2^*τ*(*ε*)*N*(*ε*) where *N* is the density of states and *v* is the group velocity, computed at 300 K and an electron concentration of 5.5 × 10^16^ cm^−3^ (Fig. 3b, c). Impurity scattering dominates at the conduction band edge but diminishes quickly as energy increases. At energies above *ω*_po_ of the band minimum (above the phonon emission threshold), polar-optical interactions are two orders of magnitude stronger than any other competing mechanism and act as the primary limiting factor for electron mobility, in agreement with the experimental findings of ref. ^65^ and DFPT + Wannier calculations^42^. In contrast, the mobility calculated using a constant relaxation time of *τ* = 10 fs—a value on the higher end of that typically employed in screening studies^25,26,28^—underestimates the mobility by a factor of 2–10 depending on the temperature, as shown in Fig. 3a. More fundamentally, the CRTA does not reproduce the correct shape of temperature dependence as depicted in Fig. 3c. The ability of our method to reproduce the qualitative temperature dependence of transport properties, as well as make good approximations of quantitative behavior (often closely in-line with more detailed theoretical methods), thus represents a major advance for improving the accuracy of high-throughput methods.

A primary goal of the present approach is to extend well-established scattering matrix elements that were formulated for isotropic materials properties to be compatible with highly anisotropic materials. To that end, we have calculated the direction-dependent hole mobilities of *Pnma* structured SnSe at a carrier concentration of 3 × 10^17^ cm^−3^, with the results compared to Hall measurements in Fig. 3d. Single-crystal SnSe has recently attracted significant attention as a thermoelectric material. Due to its layered structure, SnSe exhibits anisotropic transport properties, with the highest thermoelectric performance observed along the *b* axis^66^. Our calculations reproduce the strong directional dependence in transport measurements, in which the mobility parallel to the layers (along *b* and *c*) is almost an order of magnitude larger than that perpendicular to the layers (along *a*). Our mobility results agree remarkably well with the considerably more computationally expensive electron–phonon calculations performed using DFPT+Wannier and *G*_0_*W*_0_ band structures^41^ (Supplementary Fig. 8). We note that additional anisotropy in the mobility between the *b* and *c* directions has been observed in high-temperature experimental measurements^66^. In both our calculations and DFPT + Wannier, however, the mobility along *b* and *c* are almost the same for temperatures above 300 K^41^. The discrepancy against the experiment is thought to derive from the use of a Hall factor *r*_*H*_ of unity when extracting the carrier concentrations needed to compute mobility^41^ (further analysis is provided in the Supplementary Discussion). As we demonstrate in Supplementary Fig. 7, access to band and **k**-dependent lifetimes can further be used to calculate electron linewidths that are qualitatively comparable to those measured through techniques such as angle-resolved photoemission spectroscopy (ARPES)^67^.

### Electron mobility and Seebeck coefficient across many systems

**Fig. 4:**
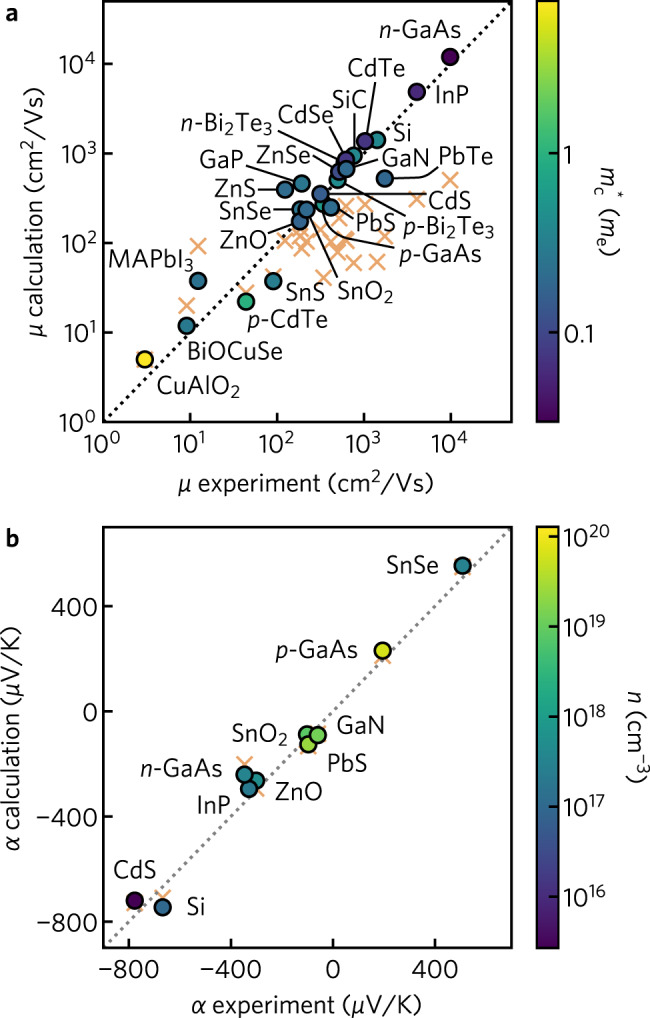
Calculated mobility and Seebeck coefficient against experiment. **a** Comparison of carrier mobilities, *μ*, at 300 K between calculations and experiments, with points colored by the conductivity effective mass ${m}_{c}^{* }$. **b** Comparison of Seebeck coefficients, *α*, at 300 K between calculations and experiments, with points colored by the majority carrier concentration *n*. For Si and CdS we compare directly to the diffusive component of Seebeck coefficient only. CH_3_NH_3_PbI_3_ has been abbreviated as MAPbI_3_. In (**a**) and (**b**), orange crosses indicate results computed using a constant relaxation time of 10 fs. Detailed temperature and carrier concentration results for each material are provided in Supplementary Figs. 8 and 11.

To demonstrate the generality of our approach, we investigate the transport properties of 21 semiconductors ranging from 2 to 48 atoms in their primitive unit cells. To highlight the compatibility of the method with high-throughput computations, all inputs (eigenvalues, wave functions, materials parameters) are obtained from density functional theory (DFT) using low-cost exchange-correlation functionals (see the “Methods” section). All such materials parameters are listed in Supplementary Table 4. Results are compared to transport measurements on high purity single-crystalline samples to minimize the effects of grain boundaries and crystallographic defects. Further details on the calculation methodology and selection of reference data are provided in the Supplementary Methods. The materials span multiple chemistries, doping polarities, and band structure types including anisotropic and multiband systems, and comprise: (i) conventional semiconductors, Si, GaN, GaP, GaAs, InP, ZnS, ZnSe, CdS, CdSe, and SiC; (ii) the thermoelectric candidates SnS, SnSe, PbTe, Bi_2_Te_3_, and BiCuOSe; (iv) photovoltaic absorbers PbS and CdTe; and (iii) transparent conductors, SnO_2_, ZnO, and CuAlO_2_. Our dataset also includes the relatively complex CH_3_NH_3_PbI_3_ hybrid perovskite containing 48-atoms. In Fig. 4a we compare calculated mobility against experimental measurements for all 21 materials in our dataset. Calculations were performed using the experimentally determined carrier concentrations at a temperature of 300 K. Results regarding the temperature and carrier concentration dependence of mobility for all materials (calculated, experimental, and comparison with CRTA) is provided in Supplementary Figs. 8 and 9 and include the breakdown of mobility by scattering type. These plots represent a comprehensive test of our approach, across many materials, not only at the single condition plotted in Fig. 4a but when conditions are varied.

The calculated mobilities agree closely with experiment across all materials, covering several orders of magnitude from ZnO (180 cm^2^/Vs) to *n*-type GaAs (${\mu }_{\exp }={\,}$2.1 × 10^4^ cm^2^/Vs). Notably, the calculated mobility (Spearman rank coefficient against experiment *r*_s_ = 0.93) improves significantly on results obtained using a constant relaxation time of *τ* = 10 fs (*r*_s_ = 0.52). As we demonstrate in Supplementary Fig. 14, our method also improves the dependence of mobility on temperature, obtaining a mean squared error (MSE) of 0.20 consistent with DFPT + Wannier (MSE = 0.19) and dramatically more accurate than a constant relaxation time (MSE = 3.2). We note that more reasonable results can be obtained in the constant relaxation time approximation by fitting the lifetime to experimental measurements. However, in most cases, experimental measurements will not be available and thus this approach cannot easily be applied to new or uncharacterized systems. One alternative is to linearly scale the constant lifetime by the temperature according to ${\tau }_{T}=\tau \frac{300}{T}$ ^68^. As demonstrated in Supplementary Figure 9, this results in a considerable improvement in the temperature dependence of mobility, reducing the mean squared error from 3.2 to 0.9. By definition, however, this does not impact the mobility at 300 K and thus does not change the constant lifetime results presented in Fig. 4a.

Our approach demonstratesgreater deviation from experiment in materials with smaller mobilities such as *p*-CuAlO_2_ (3 cm^2^/Vs in *a*-*b* plane), where a local hopping mechanism is proposed to compete with band transport^69^, and *p*-CdTe in which spin-orbit coupling (SOC) is known to dramatically impact the scattering rates at the valence band edge^70^ but was not included in our calculations. Additional deviation is observed for *n*-ZnS, where the calculated mobility is almost a factor of 4 larger than Hall measurements. We find this overestimation is largely due to the underestimation of the conduction band effective mass arising from the use of the PBE exchange-correlation functional (${m}_{c}^{* ,{\rm{PBE}}}=$ 0.16 *m*_*e*_) when compared to experiment (${m}_{c}^{* ,\exp }=$ 0.22 *m*_*e*_)^71^. As we detail in Supplementary Fig. 10, calculations performed using the hybrid HSE06 functional result in a larger effective mass (${m}_{c}^{* ,{\rm{HSE}}}=$ 0.20 *m*_*e*_) and improved agreement with the experimental mobility. Conversely, the mobility of *n*-type PbTe and *p*-type SnS are underestimated relative to the experiment. In both compounds, this originates from an overestimation of the effective mass. For example, in PbTe, HSE06 with the inclusion of spin-orbit coupling effects is required to reduce the effective mass from ${m}_{c}^{* ,{\rm{PBE}}}=$ 0.11 *m*_*e*_ to ${m}_{c}^{* ,{\rm{HSE+SOC}}}=$ 0.03 *m*_*e*_, in line with the experimental effective mass of 0.02 *m*_*e*_. In Supplementary Fig. 10, we demonstrate that more accurate treatment of the electronic structure significantly improves the agreement against the experiment across the full temperature range. Lastly, the deviation seen for CH_3_NH_3_PbI_3_ is likely due to the use of polycrystalline thin films in experimental measurements. As highlighted by Supplementary Fig. S8, our temperature-dependent results are in excellent agreement (within 5% at all temperatures) against fully first principles calculations performed using EPW^37^. The ability of our approach to accurately describe the electron–phonon coupling of a highly complex structure with 144 phonon-modes while remaining computationally efficient highlights its potential in high-throughput screening of transport behavior.

Accurate calculation of Seebeck coefficients is of primary interest in the prediction and analysis of thermoelectric materials. In Fig. 4b we compare calculated Seebeck coefficients against those obtained experimentally at 300 K. A comparison of the temperature dependence of the Seebeck coefficient for all materials is provided in Supplementary Fig. 11. We see reasonable agreement against experiment across the full range of materials, for both *p*- and *n*-type samples, corresponding to positive and negative Seebeck coefficients, respectively. In Supplementary Fig. 12, we demonstrate that use of the HSE06 hybrid functional can further improve the agreement against the experiment for *n*-type GaAs. We note that for Si and CdS we compare directly to the diffusive component of Seebeck coefficient only, ignoring the effects of phonon drag which contribute substantially even at room temperature^72–75^. The Seebeck coefficient displays a weaker dependence on electron lifetimes than mobility and conductivity and so is often treated within the CRTA (in which case the specific relaxation time cancels in the equations).^27^. Figure 4b indicates that this approximation is often justified due to the relatively small disagreements between constant relaxation time and mode-dependent relaxation time results, in-line with previous comparisons of CRTA against experimental data^76^.

## Discussion

A key motivation in the development of the present approach is the opportunity to obtain accurate carrier lifetimes at minimal computational expense. Ideally, the method should be cheap enough to permit the calculation of transport properties for thousands of compounds in a high-throughput manner as well as large and complex materials. This would allow for reliable screening of materials for functional applications as well as enable investigations of systems with larger unit cells and more complex crystal structures. We stress that an expensive DFPT calculation is not required to obtain the matrix elements unlike methods such as EPW^30^, PERTURBO^35^, and EPIC STAR^45^. In our approach, the primary computational expense is the calculation of first-principles inputs, particularly the dielectric constant as detailed in Supplementary Table 2. However, due to our use of the relatively low-cost PBE exchange-correlation functional all inputs (electronic structure, Γ-point phonon frequencies, elastic constants, dielectric constants and piezoelectric tensor) can be obtained with moderate computational requirements (generally <50 core hours to compute all properties, see Supplementary Table 2). The calculation of transport properties takes even less time; the results for each material presented in this work were computed in under an hour on a personal laptop—further timing analysis, indicating the breakdown for different routines in the code, is presented in Supplementary Fig. 4. In addition, many of the materials properties required to calculate the scattering matrix elements are already available in computational materials databases. For example, at the time of writing the Materials Project contains over 3300 piezoelectric tensors, 7100 dielectric constants and phonon frequencies, and over 13,000 elastic constants^62,77,78^. Accordingly, our approach is well suited for the large-scale analysis of transport properties. To that end, we have made available a Python implementation of the method called Ab initio Scattering and Transport (AMSET) at https://github.com/hackingmaterials/amset. Our goal is for this software to complement higher-level methods, such as EPW^30^ and PERTURBO^35^, which are state-of-the-art but significantly more computationally demanding. A schematic overview of the package, indicating the inputs, outputs and command-line tools is given in Supplementary Fig. 3.

We stress that all electronic dispersions and wave functions were computed using the PBE functional which tends to over-delocalise electronic states and underestimate effective masses. In most cases, the calculated mobility is overestimated compared to the experiment, suggesting that the use of higher-level methods such as hybrid DFT or GW will be beneficial. In addition, there are several limitations of the current approach that may be addressed in a future release. In particular, optical deformation potential scattering is not treated, the symmetry of phonon modes is not used for filtering scattering events, and our matrix elements are not yet suitable for metals.

In conclusion, we introduced a method for calculating electron lifetimes and transport properties of semiconductors and insulators. Our method extends isotropic scattering matrix elements to support highly anisotropic materials and relies on a Brillouin zone integration scheme that overcomes the need for highly dense **k**-point sampling. The present approach offers similar accuracy to state-of-the art methods at ~1/500th the computational cost and relies only on inputs that can be obtained from low-cost ab initio methods and that are routinely available in computational materials science databases. Furthermore, the agreement of mobility against experiment (Spearman rank coefficient *r*_s_ = 0.93) improves significantly on other low-cost methods such as a constant relaxation time approximation (*r*_s_ = 0.52) and temperature dependence is accurately captured. We expect that our method will enable accurate screening of transport properties in high-throughput computational workflows.

## Methods

All DFT calculations were performed using the Perdew–Burke–Ernzerhof (PBE) exchange-correlation functional^79^ as implemented in the Vienna ab initio Simulation Package (VASP)^80,81^. Materials parameters, including elastic constants, dielectric tensors, deformation potentials, and phonon frequencies are listed in Supplementary Table 4. Calculations were performed in a plane-wave basis set with scalar relativistic psueodpoentials and with the interactions between core and valence electrons described using the projector augmented-wave method (PAW)^82,83^. The set-up, submission, and management of first-principles calculations were handled using the atomate workflow management software with the default parameters of version 0.8.3^84,85^. The plane-wave energy cutoff was set to 520 eV. Structure optimization was performed with a reciprocal **k**-point density of 64 **k**-points/Å3. The uniform non-self-consistent calculations used as input to the scattering calculations were run with a reciprocal **k**-point density of 1000 **k**-points/Å3. Band gaps are corrected using a scissor operation to match those calculated by the Heyd–Scuseria–Ernzerhof (HSE06) hybrid functional^86,87^. Piezeoelectric constants, and static and high-frequency dielectric constants were computed using DFPT based on the method developed and by Baroni and Resta^88^ and adapted to the PAW formalism by Gajdoš et al.^89^. Elastic constants were obtained through the stress-strain approach detailed in ref. ^78^. Spin-orbit interactions were included for calculations on CH_3_NH_3_PbI_3_ as they were necessary to obtain the correct band ordering at the conduction band minimum. A comparison of the experimental and HSE06 band gaps, along with initial and interpolated **k**-point meshes are provided in Supplementary Table 5. All timing information (first-principles inputs and transport properties) displayed in Fig. 2a was obtained on a Intel Xeon Haswell processor E5-2698 v3 @ 2.3 GHz, except the EPW timing for NbFeSb. EPW timing information for NbFeSb was reported in ref. ^39^ without specifying the processor type, so we have assumed a 1:1 correspondence in core performance.

## Supplementary information

Supplementary Information

Description of Additional Supplementary Files

Supplementary Software 1

## Data Availability

Source data are provided with this paper. Additional temperature-dependent datasets and input files generated and analysed in the current study are available from ref. ^94^. Source data are provided with this paper.

## Source data

Source Data

## Code availability

A Python implementation of the method called Ab initio Scattering and Transport (AMSET) is made available at https://github.com/hackingmaterials/amset, archived in ref. ^95^, and provided as supplementary software 1.
