# Supplementary material for: Efficient calculation of carrier scattering rates from first principles
Source: Nat Commun. 2021 Apr 13;12:2222. doi: 10.1038/s41467-021-22440-5 (PMC8044096; doi:10.1038/s41467-021-22440-5)
Supplement: Supplementary file 1 — Supplementary Information [file 41467_2021_22440_MOESM1_ESM.pdf]

Efficient calculation of electron scattering rates from first principles

Alex M. Ganose,[1] Junsoo Park,[1] Alireza Faghaninia,[1] Rachel Woods-Robinson,[1, 2] Kristin A. Persson,[2, 3] and Anubhav Jain[1]

[1]*Energy Technologies Area, Lawrence Berkeley National Laboratory, Berkeley, California 94720, USA*
[2]*Department of Materials Science and Engineering, University of California Berkeley, California 94720, United States*
[3]*Molecular Foundry, Energy Sciences Area, Lawrence Berkeley National Laboratory, Berkeley, California 94720, USA*

(Dated: March 8, 2021)

I. SUPPLEMENTARY METHODS

A. Theoretical Framework

1. *Linearized Boltzmann transport equation*

Electron mobility, μ_e, can be computed through the linearized Boltzmann transport equation (BTE) [1–4], given for electrons as

$$\mu_{e,\alpha\beta} = \frac{-1}{n_e \Omega} \sum_{n \in \mathrm{cb}} \int \frac{\mathrm{d}\mathbf{k}}{\Omega_\mathrm{BZ}} v_{n\mathbf{k},\alpha} \partial_{E_\beta} f_{n\mathbf{k}}, \tag{1}$$

where α and β denote Cartesian coordinates, n_e is the electron concentration, Ω and Ω_BZ are the volumes of the unit cell and first Brillouin zone, respectively, $v_{n\mathbf{k},\alpha}$ is the group velocity of band index n and wave vector $\mathbf{k}$, "cb" stands for conduction bands, and $\partial_{E_\beta} f_{n\mathbf{k}}$ is the perturbation to the Fermi–Dirac distribution by an electric field $\mathbf{E}$. The Fermi–Dirac distribution is given by

$$f^0_{n\mathbf{k}} = \frac{1}{\exp\left[(\varepsilon_{n\mathbf{k}} - \varepsilon_\mathrm{F})/k_\mathrm{B}T\right] + 1}, \tag{2}$$

where $\varepsilon_{n\mathbf{k}}$ is the energy of state $n\mathbf{k}$, ε_F is the Fermi level, k_B is the Boltzmann constant, and T is temperature. The perturbation to the equilibrium Fermi–Dirac distribution is given by the self-consistent solution of

$$\begin{aligned}\partial_{E_\beta} f_{n\mathbf{k}} = e\frac{\partial f^0_{n\mathbf{k}}}{\partial \varepsilon_{n\mathbf{k}}} v_{n\mathbf{k},\beta}\tau_{n\mathbf{k}} + \frac{2\pi\tau_{n\mathbf{k}}}{\hbar}\sum_m \int \frac{\mathrm{d}\mathbf{q}}{\Omega_\mathrm{BZ}} |g_{nm}(\mathbf{k},\mathbf{q})|^2 \\ \times [(n_\mathbf{q} + 1 - f^0_{n\mathbf{k}})\delta(\Delta\varepsilon^{nm}_{\mathbf{k},\mathbf{q}} + \hbar\omega_\mathbf{q}) \\ + (n_\mathbf{q} + f^0_{n\mathbf{k}})\delta(\Delta\varepsilon^{nm}_{\mathbf{k},\mathbf{q}} - \hbar\omega_\mathbf{q})]\partial_{E_\beta} f_{m\mathbf{k}+\mathbf{q}},\end{aligned} \tag{3}$$

where $\tau_{n\mathbf{k}}$ is the electron lifetime, δ is the Dirac delta function, $\Delta\varepsilon^{nm}_{\mathbf{k},\mathbf{q}} = \varepsilon_{n\mathbf{k}} - \varepsilon_{m\mathbf{k}+\mathbf{q}}$, $\hbar$ is the reduced Planck constant, and $n_\mathbf{q}$ is the Bose–Einstein occupation. The matrix elements $g_{nm}(\mathbf{k},\mathbf{q})$ give the probability of scattering from an initial state $n\mathbf{k}$ to final state $m\mathbf{k}+\mathbf{q}$ via a phonon with wave vector $\mathbf{q}$ and frequency $\omega_\mathbf{q}$.

The primary complexity in the Boltzmann transport equation results from the dependence of the linear response coefficients $\partial_{E_\beta} f_{n\mathbf{k}}$ of state $n\mathbf{k}$ on all other states $m\mathbf{k}+\mathbf{q}$. Accordingly, there are several common approximations to the BTE that can significantly reduce the computational cost. The *momentum relaxation time approximation* (MRTA) makes two simplifications: (i) Firstly, the linear response coefficients are presumed to only act in the direction of the band velocity, such that the electron lifetimes will be *scalar* quantities [2, 4]. (ii) Secondly, the probability of scattering from state $n\mathbf{k}$ to $m\mathbf{k}+\mathbf{q}$ is assumed to be the same as scattering from state $m\mathbf{k}+\mathbf{q}$ to $n\mathbf{k}$. The result is that the effects of back scattering are accounted for by a geometrical factor resulting from the electronic group velocities. The resulting expression for $\tau^{-1}_{n\mathbf{k}}$ can be written

$$\tau^{-1}_{n\mathbf{k}} = \sum_m \int \frac{\mathrm{d}\mathbf{q}}{\Omega_\mathrm{BZ}} \left[1 - \frac{\mathbf{v}_{n\mathbf{k}} \cdot \mathbf{v}_{m\mathbf{k}+\mathbf{q}}}{|\mathbf{v}_{n\mathbf{k}}|^2}\right] \tau^{-1}_{n\mathbf{k}\to m\mathbf{k}+\mathbf{q}}, \tag{4}$$

where $\tau^{-1}_{n\mathbf{k}\to m\mathbf{k}+\mathbf{q}}$ is the partial decay rate for scattering from initial state $n\mathbf{k}$ to final state $m\mathbf{k}+\mathbf{q}$. In this approximation, Supplementary Eq. (1) can be rewritten

$$\mu^\mathrm{MRTA}_{e,\alpha\beta} = \frac{e}{n_e\Omega}\sum_{n\in\mathrm{cb}} \int \frac{\mathrm{d}\mathbf{k}}{\Omega_\mathrm{BZ}} \frac{\partial f^0_{n\mathbf{k}}}{\partial \varepsilon_{n\mathbf{k}}} v_{n\mathbf{k},\alpha} v_{n\mathbf{k},\beta}\tau_{n\mathbf{k}}. \tag{5}$$

A further simplification can be made by ignoring the effcts of scattering back into the state $n\mathbf{k}$ entirely. This corresponds to neglecting the second term on the right-hand side of Supplementary Eq. (3) or setting the geometric factor in the square bracket of Supplementary Eq. (4) to 1. In this approach, termed the *self-energy relaxation time approximation* (SERTA) [3], the electron lifetimes can be obtained according to

$$\tau^{-1}_{n\mathbf{k}} = \sum_m \int \frac{\mathrm{d}\mathbf{q}}{\Omega_\mathrm{BZ}} \tau^{-1}_{n\mathbf{k}\to m\mathbf{k}+\mathbf{q}}, \tag{6}$$

and the mobility calculated in the same manner as Supplementary Eq. (5).

The partial decay rates of Supplementary Eqs. (4) and (6) can be obtained through Fermi's golden rule. In the present work, we implement two classes of scattering: (i) inelastic scattering which occurs via emission or absorption of a phonon and (ii) perfectly elastic scattering in which no energy is gained or lost. In the case of inelastic scattering, the partial decay rate can be written [5, 6]

$$\begin{aligned}\tau^{-1}_{n\mathbf{k}\to m\mathbf{k}+\mathbf{q}} = \frac{2\pi}{\hbar}|g_{nm}(\mathbf{k},\mathbf{q})|^2 \\ \times [(n_\mathbf{q} + 1 - f^0_{m\mathbf{k}+\mathbf{q}})\delta(\Delta\varepsilon^{nm}_{\mathbf{k},\mathbf{q}} - \hbar\omega_\mathbf{q}) \\ + (n_\mathbf{q} + f^0_{m\mathbf{k}+\mathbf{q}})\delta(\Delta\varepsilon^{nm}_{\mathbf{k},\mathbf{q}} + \hbar\omega_\mathbf{q})],\end{aligned} \tag{7}$$

Supplementary Table 1. Summary of scattering mechanisms

Name	Required properties	Type	Refs.
Ionized impurity	Static dielectric	Elastic	[7, 8]
Acoustic deformation potential	Deformation potential, elastic constant	Elastic	[9–12]
Piezoelectric acoustic	Piezoelectric constant	Elastic	[13–15]
Polar optical phonon	Static and high-frequency dielectric, phonon frequency	Inelastic	[16]

where the $-\hbar\omega_{\mathbf{q}}$ and $+\hbar\omega_{\mathbf{q}}$ terms correspond to scattering by emission and absorption of a phonon, respectively. The dependence of $\tau^{-1}_{n\mathbf{k}\to m\mathbf{k}+\mathbf{q}}$ on the occupation of state $m\mathbf{k}+\mathbf{q}$ and the observation that $f_{m\mathbf{k}+\mathbf{q}} \neq f_{n\mathbf{k}}$ reveals that inelastic scattering is not commutative — i.e., $\tau^{-1}_{n\mathbf{k}\to m\mathbf{k}+\mathbf{q}} \neq \tau^{-1}_{m\mathbf{k}+\mathbf{q}\to n\mathbf{k}}$. We note that for spin polarized materials, scattering only occurs between states in the same spin channel — i.e., there are no interactions between spin-up and spin-down electrons.

For elastic scattering, Supplementary Eq. (7) reduces to

$$\tau^{-1}_{n\mathbf{k}\to m\mathbf{k}+\mathbf{q}} = \frac{2\pi}{\hbar}|g_{nm}(\mathbf{k},\mathbf{q})|^2\delta\big(\Delta\varepsilon^{nm}_{\mathbf{k},\mathbf{q}}\big). \tag{8}$$

In contrast to inelastic scattering, elastic processes do not depend on the occupation of state $m\mathbf{k}+\mathbf{q}$. Accordingly, $\tau^{-1}_{n\mathbf{k}\to m\mathbf{k}+\mathbf{q}} = \tau^{-1}_{m\mathbf{k}+\mathbf{q}\to n\mathbf{k}}$ and a primary assumption of the MRTA is satisfied. For this reason, we treat elastic scattering processes under the MRTA, whereas inelastic scattering processes are treated in the SERTA.

2. *Scattering matrix elements*

The general form of the quantum mechanical scattering matrix elements in Supplementary Eqs. (3), (4), and (6) is

$$g_{nm}(\mathbf{k},\mathbf{q}) = \langle m\mathbf{k}+\mathbf{q}|\Delta_{\mathbf{q}}V|n\mathbf{k}\rangle \tag{9}$$

where $\Delta_{\mathbf{q}}V$ is an electronic perturbation associated with a scattering process [6]. In the present work we calculate matrix elements within the Born approximation [17]; namely, the electronic perturbation is assumed to only weakly impact the wave function of the final state $m\mathbf{k}+\mathbf{q}$. The scattering matrix elements considered in this work and the materials parameters needed to calculate them are summarized in Supplementary Table 1.

G-vector summation The matrix elements include a sum over reciprocal lattice vectors **G**. In this work, we restrict the summation to only include a single reciprocal lattice vector, $\mathbf{G} = [0,0,0]$, such that only phonons within the first Brillouin zone are considered but Umklapp scattering processes (with respect to the electronic Brillouin zone) are taken into account.

Impurity scattering The inverse screening length β, required in the calculation of the ionized impurity matrix element, is given by

$$\beta^2 = \frac{e^2}{\epsilon_{\mathrm{s}} k_{\mathrm{B}} T \Omega}\sum_n \int f^0_{n\mathbf{k}}(1-f^0_{n\mathbf{k}})\,\mathrm{d}\mathbf{k}\,, \tag{10}$$

where $1/\beta$ corresponds to the Debye length and Thomas–Fermi screening length for non-degenerate and degenerate doping regimes, respectively [18].

3. *Transport properties*

Electronic transport properties — namely, conductivity, Seebeck coefficient, and electronic component of thermal conductivity — are calculated through the Onsager coefficients [19, 20]. The spectral conductivity, defined as

$$\Sigma_{\alpha\beta}(\varepsilon) = \sum_n \int \frac{\mathrm{d}\mathbf{k}}{8\pi^3} v_{n\mathbf{k},\alpha} v_{n\mathbf{k},\beta} \tau_{n\mathbf{k}} \delta(\varepsilon - \varepsilon_{n\mathbf{k}}), \tag{11}$$

is used to compute the moments of the generalized transport coefficients

$$\mathcal{L}^n_{\alpha\beta} = e^2 \int \Sigma_{\alpha\beta}(\varepsilon)(\varepsilon_{\mathrm{F}}-\varepsilon)^n \left[-\frac{\partial f^0}{\partial \varepsilon}\right]\mathrm{d}\varepsilon\,, \tag{12}$$

where ε_{F} is the Fermi level at a certain doping concentration and temperature T. Electrical conductivity (σ), Seebeck coefficient (S), and the charge carrier contribution to thermal conductivity (κ) are obtained as

$$\sigma_{\alpha\beta} = \mathcal{L}^0_{\alpha\beta}, \tag{13}$$

$$S_{\alpha\beta} = \frac{1}{eT}\frac{\mathcal{L}^1_{\alpha\beta}}{\mathcal{L}^0_{\alpha\beta}}, \tag{14}$$

$$\kappa_{\alpha\beta} = \frac{1}{e^2 T}\left[\frac{(\mathcal{L}^1_{\alpha\beta})^2}{\mathcal{L}^0_{\alpha\beta}} - \mathcal{L}^2_{\alpha\beta}\right]. \tag{15}$$

B. Computational Framework

1. *Brillouin-zone interpolation and integration*

As described in the main text, we employ a combined Fourier-linear interpolation scheme when calculating scattering and transport properties. Electronic eigenvalues — calculated using density functional theory (DFT) on a coarse **k**-point mesh — are Fourier interpolated onto a denser mesh. Fourier interpolation is performed using the BOLTZTRAP2 software [22, 23] which enforces symmetry using star functions and employs the Shankland algorithm to ensure that both quasi-particle

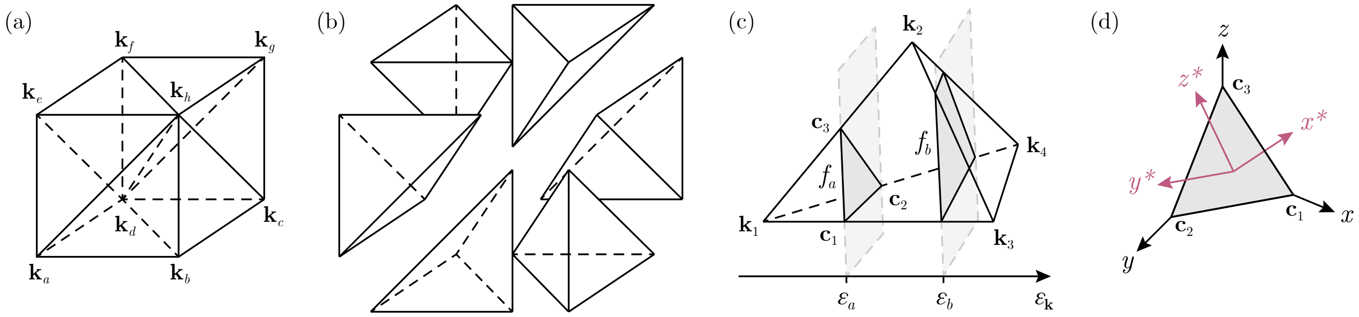

Supplementary Figure 1. Schematic of the linear-tetrahedron method. (a) A $2 \times 2 \times 2$ **k**-point submesh can be broken up into (b) six tetrahedra. Adapted from Supplementary Ref. [21]. (c) The constant energy surfaces (light gray planes) defined by ε_a and ε_b intersect the tetrahedron to produce the cross sections f_a (dark gray triangle) and f_b (dark gray quadrangle). The triangular cross section f_a is defined by the points $\mathbf{c}_1$, $\mathbf{c}_2$, and $\mathbf{c}_3$. The **k**-points at the tetrahedron vertices have been numbered according to increasing energy, i.e., $\varepsilon_{\mathbf{k}_1} < \varepsilon_{\mathbf{k}_2} < \varepsilon_{\mathbf{k}_3} < \varepsilon_{\mathbf{k}_4}$. (d) Coordinate transformation from initial basis (black arrows) to transformed basis (pink arrows) that maps the cross section onto a 2D plane. The x^* coordinates of all points on the cross section are zero.

energies and their derivatives (group velocities) are exactly reproduced [24–26]. This approach aims to minimise the roughness function proposed in Supplementary Ref. [27].

Scattering rates are calculated on the Fourier interpolated **k**-point mesh. When calculating the partial decay rate, scattering is limited to the constant energy surface defined by $\varepsilon = \varepsilon_{n\mathbf{k}}$ in the case of elastic processes [Supplementary Eq. (8)] and $\varepsilon = \varepsilon_{n\mathbf{k}} \pm \hbar\omega_{\mathbf{q}}$ for inelastic processes [Supplementary Eq. (7)]. Note that, in our implementation of polar optical phonon scattering we rely on a single dispersionless phonon mode, whose energy $\hbar\omega_{\mathrm{po}}$ is independent of **q**. Due to finite **k**-point sampling, it is common replace the delta function in Supplementary Eqs. (7) and (8) by Gaussian or Lorentzian functions with finite broadening. This procedure has the effect that the calculated lifetimes will depend on the chosen broadening parameter.

An alternative approach is to employ the linear tetrahedron method to analytically integrate the scattering rates across the constant energy surface [21, 28]. In this method, the Brillouin zone is divided into tetrahedra [Supplementary Figs. 1(a) and 1(b)]. For each electronic band, the eigenvalues are obtained for the **k**-points at the corners of the tetrahedra. The constant energy surface defined by $\varepsilon_{n\mathbf{k}}$ intersects a tetrahedron if $\varepsilon^{\mathrm{min}}_{\mathrm{tetra}} < \varepsilon_{n\mathbf{k}} < \varepsilon^{\mathrm{max}}_{\mathrm{tetra}}$, where $\varepsilon^{\mathrm{min}}_{\mathrm{tetra}}$ and $\varepsilon^{\mathrm{max}}_{\mathrm{tetra}}$ are the minimum and maximum energies of the tetrahedron's vertices [Supplementary Fig. 1(c)]. Computing the intersections of $\varepsilon_{n\mathbf{k}}$ with all tetrahedra gives rise to a set of tetrahedron cross-sections that define the constant energy surface. In the traditional implementation of the tetrahedron method, the integration for each tetrahedron is performed analytically after linearly interpolating the eigenvalues and matrix elements inside the tetrahedron. As we note in the main text, this approach is only valid for matrix elements that show a linear dependence on **q**. For ionized impurity scattering, where the matrix element has a $1/|\mathbf{q}|^2$ dependence, this assumption does not hold and results in severe overestimation of the scattering rate.

To overcome this limitation, we employ a modified linear-tetrahedron approach. The constant energy surface is determined in the same manner as the tetrahedron method. However, instead of analytically integrating within each tetrahedra, the tetrahedron cross sections (comprising the constant energy surface) are numerically resampled with hundreds of extra points. By only computing additional **k**-points that exactly satisfy the delta term in Supplementary Eqs. (7) and (8), this allows for "effective" **k**-point mesh densities that would be almost impossible to achieve with uniform **k**-point sampling. The scattering matrix elements are computed on the denser submesh by linear interpolation of the electronic wave functions $\psi_{n\mathbf{k}}$ and group velocities $\mathbf{v}_{n\mathbf{k}}$. We note that the scattering wave vector **q** is a geometric term that is known exactly for all points on the submesh. A primary advantage of this approach is that while the matrix elements cannot be linearly interpolated with **q**, the constituent parameters (electronic wave functions and group velocities) *are* linearly interpolatable.

In order to resample the constant energy surface, the tetrahedron cross sections are projected onto a two-dimensional plane. First, the **k**-points that define the tetrahedron cross sections are identified. These are the points at the intersection of the constant energy surface and tetrahedron boundary under the assumption that the band energies vary linearly between adjacent vertices in the tetrahedron [points labelled **c** in Supplementary Fig. 1(c)]. This results in three and four sets of **k**-points for triangular and quadrilateral cross sections, respectively, termed **C**. The first basis vector for the new coordinate system, **B**, is the vector normal to the plane of the cross section, namely

$$\mathbf{b}_1 = \frac{\mathbf{c}_2 - \mathbf{c}_1}{|\mathbf{c}_2 - \mathbf{c}_1|} \times \frac{\mathbf{c}_3 - \mathbf{c}_1}{|\mathbf{c}_3 - \mathbf{c}_1|},$$

where $\mathbf{c}_1$ and $\mathbf{c}_2$ are the coordinates of the first and second vertices defining the cross section. The second and third basis vectors are defined as

$$\mathbf{b}_2 = \frac{\mathbf{c}_2 - \mathbf{c}_1}{|\mathbf{c}_2 - \mathbf{c}_1|},$$
$$\mathbf{b}_3 = \mathbf{b}_2 \times \mathbf{b}_1,$$

The reciprocal space coordinates defining the cross section are transformed onto the new basis through

$$\mathbf{c}_i^{\text{proj}} = \mathbf{B}^{-1} \cdot \mathbf{c}_i.$$

In the new coordinate system, the first component of all coordinates will be the same, as all vertices lie on a plane. The last two components of the coordinates define a two-dimensional (2D) projection of the cross section which can be resampled through numerical quadrature schemes [Supplementary Fig. 1(d)]. In the present work, we employ degree 50 Xiao–Gimbutas (containing 453 sample points, [29]) or Festa–Sommariva quadratures (454 points, [30]) for resampling triangular and quadrilateral tetrahedron cross-sections, respectively. Resampling, including generating sample points and integration weights w_i^{res}, is performed using the QUADPY software [31]. The set of sample points are transformed back into the original coordinate system through

$$\mathbf{c}_i = \mathbf{B} \cdot \mathbf{c}_i^{\text{proj}}.$$

The contribution of each tetrahedron to the constant energy surface is weighted by a geometric factor that accounts for the tetrahedron's shape in four dimensional space (reciprocal coordinates and energy space) [28]. Using the triple $\mathbf{r}_i$ contragradient to vertices of the tetrahedron $\mathbf{k}_i$

$$\mathbf{r}_i \mathbf{k}_i = \delta_{ij},$$
$$\mathbf{r}_1 = \frac{\mathbf{k}_3 \times \mathbf{k}_4}{\Omega},$$
$$\mathbf{r}_2 = \frac{\mathbf{k}_4 \times \mathbf{k}_2}{\Omega},$$
$$\mathbf{r}_3 = \frac{\mathbf{k}_2 \times \mathbf{k}_3}{\Omega},$$

where the $\mathbf{k}$-points have been numbered according to increasing energy, i.e., $\varepsilon_{\mathbf{k}_1} < \varepsilon_{\mathbf{k}_2} < \varepsilon_{\mathbf{k}_3} < \varepsilon_{\mathbf{k}_4}$, the tetrahedron weight is given by [28]

$$w^{\text{tet}} = \left| \sum_{i=2}^{4} \left(\varepsilon_{\mathbf{k}_i} - \varepsilon_{\mathbf{k}_1}\right) \mathbf{r}_{i-1} \right|^{-1}.$$

We stress that this weight is distinct from the integration weights defined by Blöchl *et al.* [21] in which the contragradient cancels when averaging over all adjacent tetrahedra. The final integration weights w_i for the sample $\mathbf{k}$-point coordinates of each cross section are scaled by the tetrahedron weight to give $w_i = w_i^{\text{res}} \cdot w^{\text{tet}}$.

When evaluating the density of states

$$N(\varepsilon) = \sum_n \int \frac{\mathrm{d}\mathbf{k}}{8\pi^3} \delta(\varepsilon - \varepsilon_{n\mathbf{k}}), \tag{16}$$

and the spectral conductivity in Supplementary Eq. (11), we employ the traditional approach to the linear-tetrahedron method described by Blöchl *et al.* [21]. Specifically, we use the energy-dependent integration weights as described in Supplementary Ref. [32] and elsewhere. Unlike the partial decay rates $\tau_{n\mathbf{k}\to m\mathbf{k}+\mathbf{q}^{-1}}$, the final lifetimes $\tau_{n\mathbf{k}}$ vary smoothly across the Brillouin zone. Accordingly, use of the linear-tetrahedron method can significantly improve the convergence of transport properties without issue.

2. *Optimization of scattering calculations*

Under typically achievable carrier concentrations (10^{16} to 10^{21} cm^2/Vs) the Fermi level will sit close to either the conduction or valence band edge. Accordingly, only $\mathbf{k}$-points that lie within a few hundred meV of the band edge will contribute to electronic transport. It is therefore unnecessary to compute the electron lifetimes for all $\mathbf{k}$-points in the band structure, as most will have no impact on transport properties. From the generalized transport coefficients $\mathcal{L}$ in Eq. (12), it can be seen that each $\mathbf{k}$-point's contribution to the transport properties is scaled by a factor $(\varepsilon_{n\mathbf{k}} - \varepsilon_{\mathrm{F}})^n \left[-\partial f_{n\mathbf{k}}^0 / \partial \varepsilon_{n\mathbf{k}}\right]$, which depends entirely on the energy of the state. Accordingly, we have designed a procedure to assess which energy range is important for transport, illustrated in Supplementary Fig. 2(a). We begin by denoting the "moment-coefficient weight" as

$$w_n(\varepsilon) = (\varepsilon_{\mathrm{F}} - \varepsilon)^n \left[-\frac{\partial f^0}{\partial \varepsilon}\right], \tag{17}$$

where the indices $n = 0, 1, 2,$ correspond to the moments of $\mathcal{L}^n$ required to compute conductivity, Seebeck coefficient, and the electronic component of thermal conductivity, respectively. This is weighted by the spectral conductivity Σ^{crt} under the assumption of a constant relaxation time [i.e., Supplementary Eq. (11) with $\tau = 1$] to give

$$w_n^{\Sigma^{\text{crt}}}(\varepsilon) = |w_n(\varepsilon)| \cdot \Sigma^{\text{crt}}(\varepsilon). \tag{18}$$

Finally, we compute the normalized cumulative integral of the weights according to

$$w_n^{\text{cum}}(\varepsilon) = \frac{\int_{-\infty}^{\varepsilon} w_n^{\Sigma^{\text{crt}}}(\varepsilon')\,\mathrm{d}\varepsilon'}{\int w_n^{\Sigma^{\text{crt}}}(\varepsilon')\,\mathrm{d}\varepsilon'}. \tag{19}$$

We can then define a tuneable parameter λ than controls the minimum and maximum energy ranges within which

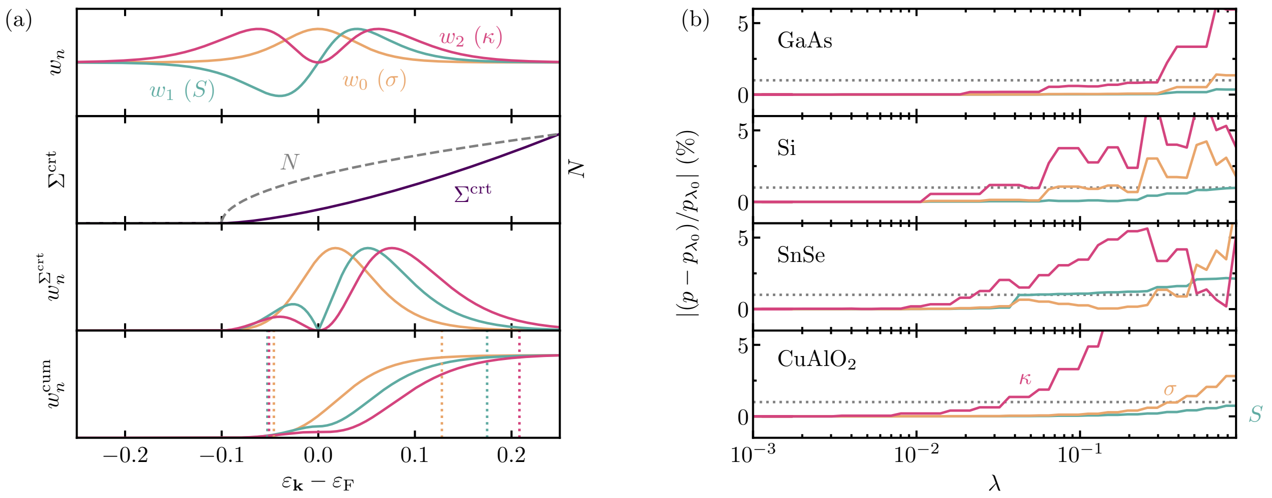

Supplementary Figure 2. (a) Procedure for obtaining the energy range in which to calculate scattering rates. The momentum coefficient weight w_n for $n = 0, 1, 2$ (top panel) is scaled by the spectral conductivity Σ^{crt} to give $w_n^{\Sigma^{\mathrm{crt}}}$. The cumulative integral of the moment weights w_n^{cum} is used to determined the energy cutoffs (bottom panel). The dashed orange, teal, and pink lines give $\varepsilon_n^{\min}$ and $\varepsilon_n^{\max}$ for $n = 0, 1, 2$, respectively at $\lambda = 0.05$. The final values of $\varepsilon^{\min}$ and $\varepsilon^{\max}$ are taken as the smallest $\varepsilon_n^{\min}$ and largest $\varepsilon_n^{\max}$ values across all moments, respectively. (b) Convergence of electronic transport properties p as a function of λ at 300 K for GaAs, Si, SnSe, and $CuAlO_2$. Absolute percentage difference from converged value $|(p - p_{\lambda_0})/p_{\lambda_0}|$ given for conductivity ($p = \sigma$, orange), Seebeck coefficient (S, teal), and electronic contribution to the thermal conductivity (κ, pink), respectively. p_{λ_0} corresponds to the value of the transport properties at $\lambda = 0$ — i.e., the scattering rates for all $\mathbf{k}$-points are calculated explicitly. Convergence within 1 % is highlighted by a dashed gray line.

to calculate the scattering rates. Namely,

$$\varepsilon_n^{\min} = \arg\min_{\varepsilon} \left| w_n^{\mathrm{cum}}(\varepsilon) - \frac{\lambda}{2} \right|, \tag{20}$$

$$\varepsilon_n^{\max} = \arg\min_{\varepsilon} \left| w_n^{\mathrm{cum}}(\varepsilon) - \left[1 - \frac{\lambda}{2} \right] \right|, \tag{21}$$

where λ can vary between 0 (in which case $\varepsilon_n^{\min}$ and $\varepsilon_n^{\max}$ will be the minimum and maximum energies in the band structure) and 1 (where $\varepsilon_n^{\min}$ and $\varepsilon_n^{\max}$ will be the same value). A value of $\lambda = 0.1$, indicates that 90 % of the integrated $w_n^{\Sigma^{\mathrm{crt}}}$ will be included in the energy range. Alternatively put, a value of $\lambda = 0.1$ results in $\varepsilon_n^{\min}$ and $\varepsilon^{\max}$ taking the energies where $w_n^{\mathrm{cum}} = 0.05$ and 0.95, respectively. The final energy range is given by $\varepsilon^{\min} = \min(\{\varepsilon_n^{\min} : n = 0, 1, 2\})$ and $\varepsilon^{\max} = \max(\{\varepsilon_n^{\max} : n = 0, 1, 2\})$. The scattering rate is only calculated for states where $\varepsilon^{\min} \leq \varepsilon_{n\mathbf{k}} \leq \varepsilon^{\max}$, with the scattering rates of the remaining states set to the average value of the rates that have been calculated explicitly. By setting λ to an appropriate value, the scattering rates for $\mathbf{k}$-points outside the energy range will not impact the transport properties.

To demonstrate the impact of λ and determine reasonable values to use in our calculations, we have investigated the convergence of the transport properties for GaAs, Si, SnSe, and $CuAlO_2$ at 300 K [Supplementary Fig. 2(b)]. The conductivity, Seebeck coefficient, and electronic contribution to the thermal conductivity of all materials are converged to within than 1 % by $\lambda = 0.02$. In most cases, the Seebeck coefficient converges the fastest, most likely due to its weaker dependence on the scattering rate. The electronic contribution to the thermal conductivity is the slowest property to converge, as expected from its reliance on a broader momentum coefficient weight. If only the conductivity or Seebeck coefficient are of interest, a much larger value of λ can be used. For example, using a λ of 0.1 converges these properties to within 1 %. In our calculations, we employ a λ of 0.05 which offers a reasonable trade-off between speed and convergence. This property is controlled in our software implementation through the `fd_tol` parameter.

3. *Software implementation*

An open-source implementation of the formalism, used to perform all calculations in this work, is released as a package called AMSET [33]. AMSET is freely available under a modified Berkeley Software Distribution (BSD) license. The current version is developed and maintained using Git and is accessible at https://hackingmaterials.lbl.gov/amset. The code can be run on both high-performance computing clusters or personal computers. AMSET is implemented in Python 3 and relies on several open-source libraries including pymatgen [34] for parsing VASP calculation outputs, BoltzTraP2 [20, 35] for Fourier interpolation of electronic eigenvalues and group velocities, SPGLIB [36] for symmetry analysis, QUADPY [31] for numerical integration, and MATPLOTLIB

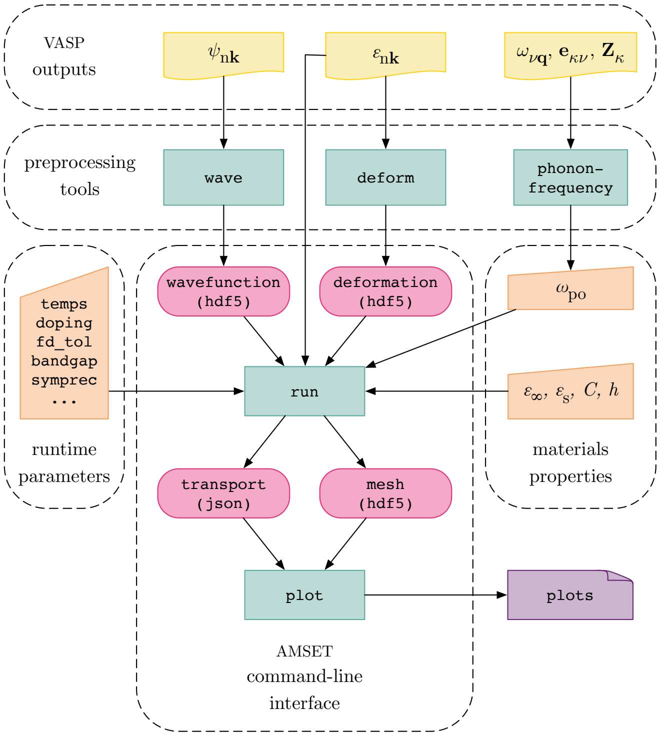

Supplementary Figure 3. Schematic of the AMSET program indicating the typical inputs and outputs, command-line tools, and program flow.

[37] for plotting. The NumPy [38] and SciPy [39] libraries are used extensively to minimize the cost of expensive matrix operations. All-electron wave function coefficients are generated from the pseudo-wave functions using the `MomentumMatrix` functionality of the PAWPYSEED package [40].

AMSET can be used through either the the command-line or a Python application programming interface (API). A typical workflow, showing computational inputs and outputs, is illustrated in Supplementary Fig. (3). The primary inputs are `vasprun.xml` and `WAVECAR` VASP output files, calculated on a uniform **k**-point mesh. Additional settings, such as the materials parameters used to calculate scattering, the doping concentrations and temperatures to consider, and accuracy settings such as `fd_tol`, can be specified in a separate file or as command-line arguments. Information on all the available settings is provided on the AMSET website. After obtaining the first principles inputs, two pre-processing steps are required. Firstly, the all-electron wave function coefficients must be extracted from the VASP `WAVECAR` file using the `wave` tool. Secondly, the "effective-phonon-frequency" should be calculated from phonon frequencies and eigenvectors, and the Born effective charges using the `phonon-frequency` tool. This process is described in more detail in Section I C 1. Scattering rates and transport properties are computed using the `run` command. The primary output is the `transport` file, which by default contains the calculated mobility, Seebeck coefficient, and electronic contribution to the thermal conductivity in the JavaScript Object Notation (JSON) format. The scattering rates, and interpolated eigenvalues and group velocities can be written to the `mesh` file with the Hierarchical Data Format version 5 (HDF5) format [41] using the `write_mesh` option. Finally, the `plot` command can be used to plot transport properties, lifetimes, and electron linewidths from the `transport` and `mesh` files. The sumo package is used for plotting band structures [42].

4. Timing analysis

A primary goal of the present approach is to be amenable to high-throughput computational workflows. To investigate the computational requirements of the AMSET package, we have illustrated the time taken to calculate the scattering rates of several of the test materials in Supplementary Fig. 4(a). All calculations were performed on a MacBook Pro with a quad core 2.9 GHz Intel Core i7 processor. The maximum time taken was 42 min for GaN, with most of the remaining materials completed in under 20 min. To understand which portions of the code are the most computationally demanding, we have broken down the results into the time taken to: (i) perform Fourier interpolation of electronic eigenvalues, (ii) compute the density of states through the tetrahedron method, (iii) obtain the scattering rates, (iv) calculate transport properties, and (v) write the output data to disk. We note, the benchmarks were performed with the `write_mesh` option enabled, so the output includes the scattering rates and interpolated band structure. In general, writing the output data takes the least amount of time relative to the other functions of the code. The breakdown for the rest of the computational steps depends strongly on the material and run time parameters, with most of the time spent calculating the scattering rates or transport properties.

To understand the scaling performance of AMSET with interpolation density, we have investigated the correlation of runtime with number of **k**-points. We find there is not a simple correlation between the total number of **k**-points and total runtime. Instead, each function of the code shows different scaling behaviour. The interpolation routines show $\mathcal{O}(n \log n)$ scaling (where n is the total number of **k**-points in the dense mesh), which is consistent with the time complexity of the fast Fourier transform algorithm. The time taken to compute scattering does not correlate well with total number of **k**-points. This is primarily as we only compute the scattering rates for the **k**-points which fall within the energy cutoffs defined by the λ parameter (see Section I B 2). In addition, we use the symmetry of the reciprocal lattice to limit our calculations to the **k**-points in the irreducible Brillouin zone (denoted $\mathbf{k}^{\text{ir}}$-points). The timing of the scattering routines correlates with the number of irreducible **k**-points that fall within the energy cutoffs, exhibiting a $\mathcal{O}(n^{1.3})$ scaling complexity. We note that,

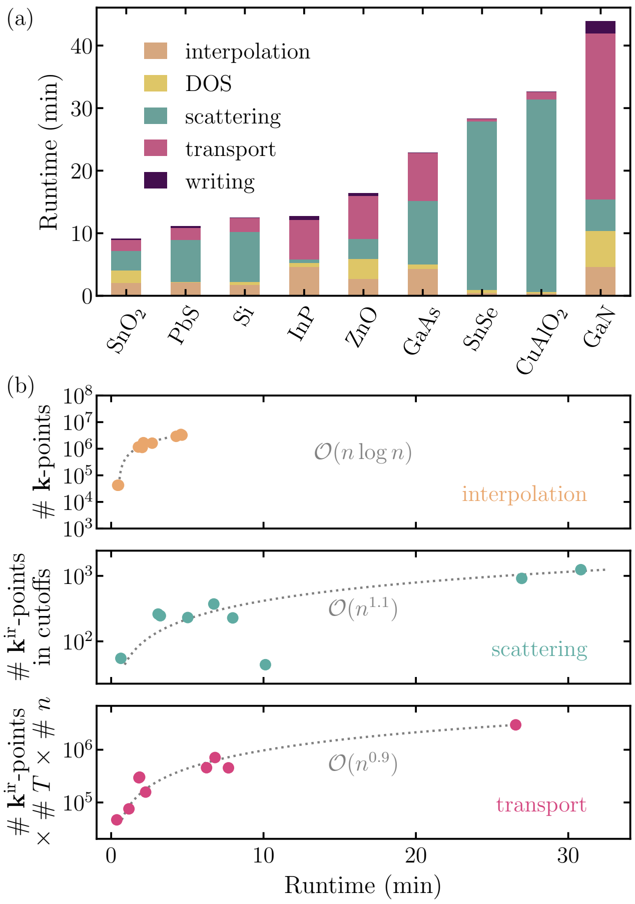

Supplementary Figure 4. Timing analysis for running AMSET on a selection of materials in the test set. Calculations performed using the materials parameters in Supplementary Table 4 and at the carrier concentrations and temperatures specified in Supplementary Table 6. (a) The total runtime for each system, broken up into the different functions of the code. (b) Correlation between time and number (denoted by #) of **k**-points for the interpolation, scattering, and transport routines. $\mathbf{k}^{\mathrm{ir}}$ indicates the **k**-points within the irreducible Brillouin zone. The number of temperatures and carrier concentrations are denoted by # T and # n, respectively. The computational complexity, provided in big O notation relative to the x-axis, is given in grey text and highlighted by dashed grey lines.

while the scattering rate is only calculated for the irreducible **k**-points within the energy cutoffs, the scattering rate for each state requires integrating the partial decay rates over the full Brillouin zone and not just the irreducible part. The time taken to compute transport properties correlates to the number the number of irreducible k-points multiplied by the number of carrier concentrations and temperatures included in the calculation, with a $\mathcal{O}(n^{0.9})$ scaling complexity. The primary expense when computing transport properties is generating the energy-dependent tetrahedron integration weights used to obtain the spectral conductivity.

Supplementary Table 2. Time required to obtain first-principles inputs given in core hours. Calculations were performed as described in the Computational Methodology. We note that the DFPT calculation listed here is performed only for a single **q**-point at Γ and is used to obtain the effective phonon frequency, static and high-frequency dielectric constants, and piezoelectric constants rather than the matrix elements $g(\mathbf{k}, \mathbf{q})$. Static+NSCF (non self-consistent field) refers to a single point calculation on the relatively dense DFT **k**-point meshes listed in Supplementary Table 5. Deformation and elastic refer to the total time required to calculate the deformation potential and elastic tensors

Material	Static+NSCF	Deformation	DFPT	Elastic	Total
GaN	1.75	15.76	13.82	32.68	64.01
GaP	1.48	13.36	8.43	10.24	33.52
GaAs	0.30	0.91	9.33	10.31	20.85
InP	0.55	4.91	9.01	4.48	18.94
ZnO	1.77	15.92	11.79	31.64	61.12
ZnS	1.46	13.11	7.11	10.21	31.88
ZnSe	1.47	13.19	7.90	10.24	32.79
CdS	1.16	10.40	14.88	18.80	45.24
CdSe	1.12	10.07	16.61	17.28	45.08
CdTe	0.93	8.33	8.11	5.84	23.22
SiC	2.57	23.16	3.23	17.69	46.66
PbS	0.53	4.80	7.31	3.67	16.31
PbTe	0.80	5.21	8.48	7.21	21.70
MAPbI$_3$[a]	6.67	40.03	901.12	65.03	1012.85
SnO_2	1.34	12.03	14.11	18.08	45.56
SnS	0.91	9.50	42.85	21.99	75.25
SnSe	1.90	17.14	48.48	24.00	91.53
Bi_2Te_3	1.55	18.08	12.12	19.11	50.86
BiCuOSe	0.79	5.64	9.88	17.56	33.87
$CuAlO_2$	1.88	16.93	25.60	34.07	78.49
Si	2.65	7.96	2.62	8.78	22.01
Ba_2BiAu	1.78	16.05	9.16	5.55	32.49
NbFeSb	1.21	12.15	5.52	6.15	25.04

[a] MA = CH_3NH_3

The total time to obtain transport properties is dominated by the calculation of the first-principles inputs (materials parameters and band structure calculation). In Supplementary Table 2, we provide the full timing information (in core hours) required to calculate all materials parameters used in this work. In Fig. (1) in the main text, we compare these times against DFPT+Wannier calculations performed using QUANTUM ESPRESSO and EPW. In Supplementary Table 3 we provide the full breakdown of the DFPT+Wannier calculations, including the references from which the timing information and mobility was extracted.

5. *Reproducing the Brooks–Herring model of impurity scattering*

A primary advantage of the present approach is that it allows, for the first time, evaluation of ionized impurity scattering in anisotropic multi-band systems. Most

Supplementary Table 3. Time required to obtain electron mobility using DFPT+Wannier, as implemented in QUANTUM ESPRESSO (DFPT to obtain $g(\mathbf{k},\mathbf{q})$ portion) and EPW (Wannier interpolation and scattering portion) in core hours. References are given to the publications in which the timing information and mobility results are reported

Material	DFPT	Scattering	Total	Refs.
Ba_2BiAu	7000	2500	9500	[43]
NbFeSb	4600	2600	7200	[44, 45]

modern computational evaluations of impurity scattering instead employ the closed-form Brooks–Herring formula [7, 8]. We will not reproduce the full derivation here but refer the reader to the excellent introduction provided in Supplementary Ref. [46]. In this approach, the scattering matrix element

$$g_{nm}(\mathbf{k},\mathbf{q}) = \frac{n_{\mathrm{ii}}^{1/2} Ze}{\epsilon_{\mathrm{s}}} \frac{1}{|\mathbf{q}|^2 + \beta^2}, \tag{22}$$

where n_{ii} and Z are the concentration and charge of the charge of the impurities, ϵ_{s} is the static dielectric constant, and β is the inverse screening length given by Supplementary Eq. (10), is analytically integrated for a single parabolic band [7, 8]. Under the assumption of complete overlap between the states the $n\mathbf{k}$ and $m\mathbf{k}+\mathbf{q}$, the resulting energy-dependent lifetime can be written

$$\tau_{\mathrm{BH}}^{-1}(\varepsilon) = \frac{n_{\mathrm{ii}} Z^2 e^4 G(b)}{\pi 16 \sqrt{2} \sqrt{m_{\mathrm{d}}^*} \epsilon_{\mathrm{s}}^2} \varepsilon^{-3/2}, \tag{23}$$

where m_{d}^* is the density of states effective mass, ϵ_0 is the vacuum permittivity, $G(b) = \ln(b+1) - b/(b+1)$, and $b = 8m_{\mathrm{d}}^* \varepsilon / \hbar^2 \beta^2$. Further integration of the energy-dependent lifetime yields the well-known Brooks–Herring mobility formula

$$\mu_{\mathrm{BH}} = \frac{128\sqrt{2\pi}\epsilon_{\mathrm{s}}^2 (k_{\mathrm{B}}T)^{3/2}}{e^3 Z^2 \sqrt{m_{\mathrm{d}}^*} n_{\mathrm{ii}} G(b)}. \tag{24}$$

To validate our implementation of ionized impurity scattering, we have generated a model parabolic electronic structure according to

$$\varepsilon_{\mathbf{k}} = \frac{\hbar^2 |\mathbf{k}|^2}{2m_{\mathrm{d}}^*}, \tag{25}$$

$$\mathbf{v}_{\mathbf{k}} = \frac{\hbar |\mathbf{k}|}{m_{\mathrm{d}}^*}, \tag{26}$$

where $\varepsilon_{\mathbf{k}}$ and $\mathbf{v}_{\mathbf{k}}$ are the energy and group velocity at wave vector $\mathbf{k}$, respectively. We calculated the ionized impurity scattering rate and resulting mobility using the AMSET package and Brooks–Herring formulas, parameterized according to $Z = 1$, $m_{\mathrm{d}}^* = 0.2\,m_0$, $\epsilon_{\mathrm{s}} = 20\,\epsilon_0$, $n_{\mathrm{ii}} = 1 \times 10^{16}\,\mathrm{cm}^{-3}$ to $1 \times 10^{19}\,\mathrm{cm}^{-3}$, and $T = 500\,\mathrm{K}$. A comparison between the two approaches is presented in Supplementary Fig. (5). Close agreement is observed for the both the mobility and carrier lifetime, indicating our approach is accurately reproducing the Brooks–Herring results.

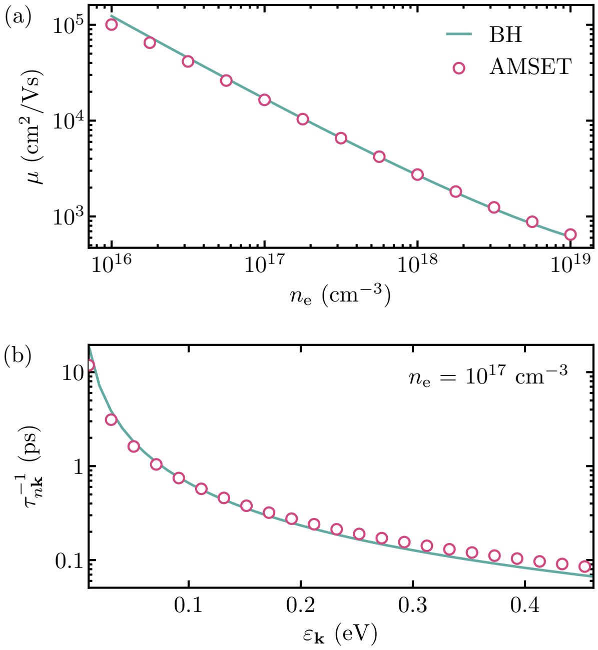

Supplementary Figure 5. Comparison of the (a) mobility and (b) carrier lifetime between AMSET and the analytical Brooks–Herring formulas for a parabolic band structure. Results calculated at a temperature of 500 K.

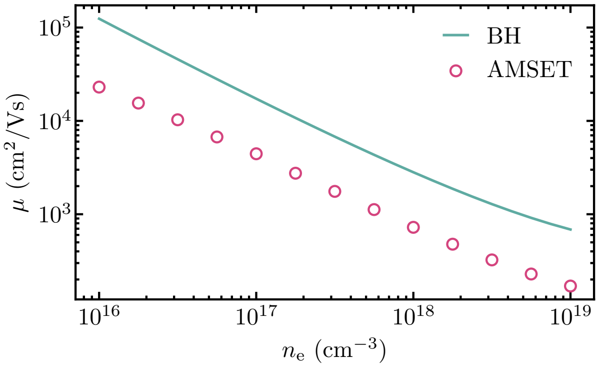

Supplementary Figure 6. Comparison of the electron mobility between AMSET and the analytical Brooks–Herring formulas for a parameterized Silicon-like band structure. Results calculated at a temperature of 500 K.

The Brooks–Herring formula is known to lead to inaccurate results for non-parabolic band structures or systems with multiple valleys. To demonstrate this, we compare our method against Brooks–Herring on an idealized Silicon band structure, as parameterized in Supplementary Refs. [47] and [48] and using the experimental effec-

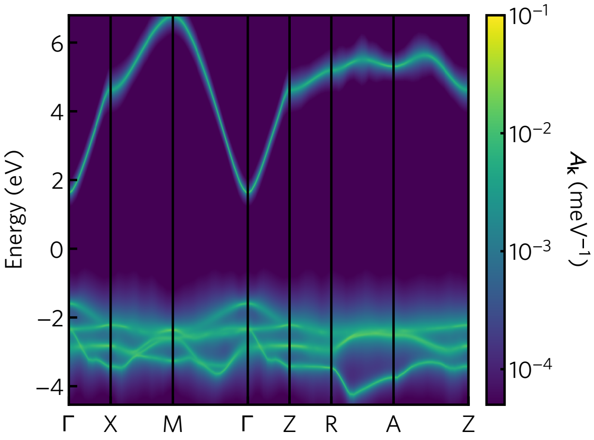

Supplementary Figure 7. Spectral band structure of SnO_2 indicating band and **k**-dependent electron linewidths calculated at 300 K.

tive masses according to

$$\varepsilon_{\mathbf{k}} = \frac{\hbar^2(k_x - k_{0,x})^2}{2m_{\parallel}^*} + \frac{\hbar^2(k_y - k_{0,y})^2}{2m_{\perp}^*} + \frac{\hbar^2(k_z - k_{0,z})^2}{2m_{\perp}^*}, \tag{27}$$

where $m_{\parallel}^* = 0.98\,m_0$, $m_{\perp}^* = 0.19\,m_0$, and $\mathbf{k}_0$ denotes the wave vectors of the conduction band minima. The Brooks–Herring mobility is calculated using the harmonic mean of the effective masses, namely $3/(m_{\parallel}^{-1,*} + 2m_{\perp}^{-1,*}) = 0.26\,m_0$. As can be seen in Supplementary Fig. (6), the Brooks–Herring mobility is considerably over estimated by almost an order of magnitude relative to the mobility computed by AMSET. This agrees well with empirical investigations into the mobility of Silicon that have noted the overestimation of the Brooks–Herring result [49].

6. *Electron linewidths*

Access to band and **k**-dependent lifetimes can further be used to calculate electron linewidths that are qualitatively comparable to those measured through techniques such as angle-resolved photoemission spectroscopy (ARPES) [50]. In Supplementary Fig. (7) we plot the spectral band structure of SnO_2 along a high symmetry Brillouin zone path, where the spectral function $A_{\mathbf{k}}(\varepsilon) = \pi^{-1}\sum_n (\tau_{n\mathbf{k}}^{-1}/2)/[(\varepsilon - \varepsilon_{n\mathbf{k}}/\hbar)^2 + (\tau_{n\mathbf{k}}^{-1}/2)^2]$ was calculated at 300 K. The spectral function provides insight into the **k**-dependence of the carrier lifetimes. States close to the conduction band edge at Γ exhibit long lifetimes (low energy broadening) due to the reduced phase space of available states for scattering. Between the Z and R high symmetry points, the lowest conduction band is relatively flat leading to large scattering rates and considerable broadening of the spectral function.

C. First-principles inputs

1. *Computational methodology*

First-principles calculations were performed using Kohn-Sham DFT [51, 52] as implemented in the Vienna *ab initio* Simulation Package (VASP) [53–55]. All *ab initio* inputs were computed within the generalized-gradient approximation (GGA) [56] using the Perdew-Burke-Ernzerhof (PBE) exchange–correlation functional [57]. Calculations were performed in a plane-wave basis set with scalar relativistic psuedopoentials and with the interactions between core and valence electrons described using the projector augmented-wave method (PAW) [58, 59]. The set-up, submission, and management of first-principles calculations was handled using the ATOMATE workflow management software with the default parameters of version `0.8.3` [60, 61]. The plane-wave energy cutoff was set to 520 eV. Structure optimization was performed using the standard pymatgen `MPRelaxSet` with a reciprocal **k**-point density of 64 **k**-points/Å^3 [34]. The uniform non-self-consistent calculations used as input to the scattering calculations were run with a reciprocal **k**-point density of 1000 **k**-points/Å^3. Spin–orbit interactions were included for calculations on $CH_3NH_3PbI_3$ as they were necessary to obtain the correct band ordering at the conduction band minimum.

Piezoelectric constants, and static and high-frequency dielectric constants were computed using density functional perturbation theory (DFPT) based on the method developed by Baroni and Resta [62] and adapted to the PAW formalism by Gajdoš *et al.* [63]. Elastic constants were obtained through the stress-strain approach detailed in Supplementary Ref. [64]. These calculations were automated using the `piezoelectric_constant`, `dielectric_constant`, and `elastic_constant` preset workflows available in ATOMATE [60]. The calculation outputs (permitivies, elastic constants) were extracted using the PYMATGEN materials science software [34].

Absolute volume deformation potentials were calculated in the manner proposed by Wei and Zunger [65]. The deformation potential describes the change in energy of the bands with applied stress. Starting from a relaxed structure, a set of distorted structures are generated as follows. The Green-Lagrange strain tensor, $S_{\alpha\beta}$, has 6 independent components (S_{11}, S_{22}, S_{33}, S_{12}, S_{13}, S_{23}), each of which is applied independently to deform the structure. For each deformed structure, the deformation potential $D_{n\mathbf{k},\alpha\beta}$ at band, n, and **k**-point, **k**, is calculated as

$$D_{n\mathbf{k},\alpha\beta} = \frac{\varepsilon_{n\mathbf{k}}^0 - \varepsilon_{n\mathbf{k}}^{\mathrm{s}} + \Delta\zeta}{S_{\alpha\beta}}, \tag{28}$$

where ε^0 and ε^{s} are the electronic energies of the bulk and strained structures, respectively, and $\Delta\zeta$ is a correction that accounts for the shift in the electrostatic reference energy between the two calculations. In this work, $\Delta\zeta$, is calculated as $\Delta\zeta = \zeta^{\mathrm{s}} - \zeta^0$, where ζ^0 and ζ^{s} are the energies of the deepest core states of the bulk and strained structures, respectively [65]. We note that, in practice, even the reference energy levels can shift upon strain, leading to a small degree of error in the deformation potentials for non-covalent crystals [66, 67]. By repeating this procedure for each of the 6 independent strain components, all elements of the 3×3 deformation potential tensor (at each band and **k**-point) can be calculated. To alleviate issues of numerical noise, we average the the deformation potentials for both contraction ($-0.5\,\%$ strain) and expansion ($+0.5\,\%$ strain) of the lattice. Furthermore, to reduce the computational requirements, the 12 independent calculations (comprising 6 independent strain components $\times$ 2 displacement strain magnitudes) are reduced using the symmetry of the bulk structure. For highly symmetric structures such as Si and GaAs, this means only 3 deformation calculations are required. We have released an open source tool `deform` as part of the AMSET package that automates the set-up of deformation calculations and the extraction of deformation potentials from VASP calculation outputs.

The "effective phonon frequency" used in the calculation of polar-optical phonon scattering was determined from the phonon frequencies $\omega_{\mathbf{q}\nu}$ (where ν is a phonon branch and $\mathbf{q}$ is a phonon wave vector) and eigenvectors $\mathbf{e}_{\kappa\nu}(\mathbf{q})$ (where κ is an atom in the unit cell). In order to capture scattering from the full phonon band structure in a single phonon frequency, each phonon mode is weighted by the dipole moment it produces according to

$$w_\nu = \sum_\kappa \left[\frac{1}{M_\kappa \omega_{\mathbf{q}\nu}} \right]^{1/2} \times \left[\mathbf{q} \cdot \mathbf{Z}^*_\kappa \cdot \mathbf{e}_{\kappa\nu}(\mathbf{q}) \right] \tag{29}$$

where $\mathbf{Z}^*_\kappa$ is the Born effective charge. This naturally suppresses the contributions from transverse-optical and acoustic modes in the same manner as the more general formalism for computing Frölich based electron-phonon coupling [68, 69]. The weight is calculated only for Γ-point phonon frequencies and averaged over the unit sphere scaled by 0.01 to capture both the polar divergence at $\mathbf{q} \to 0$ and any anisotropy in the dipole moments. The effective phonon frequency is calculated as the weighted sum over all Γ-point phonon modes according to

$$\omega_{\mathrm{po}} = \frac{\omega_{\Gamma\nu} w_\nu}{\sum_\nu w_\nu}. \tag{30}$$

We have released an open source tool `phonon-frequency` as part of the AMSET package that automates this computation from VASP calculation outputs.

2. *Computational input settings*

In this section we detail the computational input parameters used to calculate the materials properties in the VASP DFT code. We stress that in addition to the settings listed below, the accuracy of the calculated properties can depend on the exchange–correlation functional, the presence of spin–orbit interactions, and the treatment of highly correlated electrons, which must be assessed on a per material basis. To obtain accurate results, the crystal structure should first be relaxed using "tight" calculation settings including high force and energy convergence criteria. An example of the VASP settings required is:

```
ADDGRID = True
EDIFF = 1E-8
EDIFFG = -5E-4
PREC = Accurate
NSW = 100
ISIF = 3
NELMIN = 5
```

Our approach requires a `vasprun.xml` file from a "dense" uniform band structure calculation. Typically a **k**-point mesh density at least twice that needed to converge the total energy will be necessary to converge transport properties. Note, this refers to the initial DFT mesh before Fourier interpolation. In order to obtain accurate band gaps often a hybrid DFT functional such as HSE06 is required.

The wave function coefficients are required to calculate wave function overlaps. This requires the `WAVECAR` file to be written by VASP (achieved by setting `LWAVE = True`). The wave function coefficients can be extracted using the `amset wave` command. Coefficients are stored in the `wavefunction.h5` file. An example of the VASP settings required to generate the wave function information is:

```
ADDGRID = True
EDIFF = 1E-8
PREC = Accurate
NSW = 1
LWAVE = True
```

AMSET includes a tool to assist with the calculation of the deformation potentials. The initial input is a "tight" optimised structure as described above. Deformed structures are generated using the `amset deform create` command, which will generate a set of deformed `POSCAR`s each corresponding to a component of the strain tensor. Symmetry is automatically used to reduce the number of calculations needed. A single point calculation (no relaxation, i.e., `NSW = 0`) should be performed for each deformed `POSCAR` as well as the bulk (undeformed) structure. An example of the VASP settings required for the single point calculations is:

```
ADDGRID = True
EDIFF = 1E-8
PREC = Accurate
```

```
NSW = 1
ICORELEVEL = 1  # write core levels to OUTCAR
```

The deformation potentials can be calculated using the `amset deform read` command. This requires the paths to the bulk and deformation calculations as inputs. The bulk folder should be specified first, followed by the deformation folders. For example,

```
amset deform read bulk def-1 def-2 def-3
```

This will write the deformation potentials to a `deformation.h5` file in the current directory.

Static and high-frequency dielectric constants, piezoelectric constants, and the "effective polar phonon frequency" can be obtained using density functional perturbation theory. It is very important to first relax the structure using tight convergence setting. An example of the VASP settings required to perform DFPT is:

```
ADDGRID = True
EDIFF = 1E-8
PREC = Accurate
NSW = 1
IBRION = 8
LEPSILON = True
```

Note, DFPT cannot be used with hybrid exchange-correlation functionals. Instead, the `LCALCEPS` flag should be used in combination with `IBRION = 6`. The dielectric constants and polar phonon frequency can be extracted from the VASP outputs using the command `amset phonon-frequency`. This command should be run in a folder containing the `vasprun.xml` file from a DFPT calculation.

3. Materials parameters

All materials parameters were computed from first-principles in the manner described in the Computational Methodology. A summary of the materials parameters used to compute carrier scattering rates is provided in Supplementary Table 4. We have additionally employed the rigid scissor approximation such that band gaps match those calculated using the hybrid HSE06 exchange–correlation functional. Supplementary Table 5 gives the band gaps and **k**-point meshes employed in our calculations. Furthermore, we report the range of temperatures and carrier concentrations at which mobility and Seebeck coefficients are computed in Supplementary Tables 6 and 7.

4. Experimental data

In the main text, we calculate the mobility and Seebeck coefficient of 17 semiconductors and compare our results to experimental measurements. Our set of test materials spans a range of chemistries and doping-polarities and contains both isotropic and anisotropic materials. The set includes: (i) conventional semiconductors, Si, GaAs, GaN, GaP, InP, ZnS, ZnSe, CdS, CdSe, and SiC; (ii) the thermoelectric candidate SnSe; (iv) photovoltaic absorbers $CH_3NH_3PbI_3$, PbS, and CdTe; and (iii) transparent conductors, SnO_2, ZnO, and $CuAlO_2$. The reference samples are of the highest purity and crystallinity in order to minimize the mesoscopic effects of grain boundary scattering and crystallographic one-dimensional and two-dimensional defects (e.g., line dislocations, edge dislocations, and stacking faults). We favor bulk crystals over thin films (which can exhibit surface effects that impact carrier transport, e.g., strain, oxidation, off-stoichiometries, and surface dipole moments), however, in some cases we use epitaxial single crystal films. We also favor undoped or dilutely doped crystals (to less than 0.5 % at.) to avoid the formation of secondary crystal phases and degenerate doping. Lastly, we favor studies that look at a wide range of carrier concentrations and/or temperatures (greater than 300K). In all cases, experimental mobility is measured via the DC Hall effect. A summary of the reference data used in the comparisons against carrier mobility and Seebeck coefficient are provided in Supplementary Tables 6 and 7.

We note that for SnSe, significant anisotropy is seen in the measured carrier concentrations for the a and b directions [122]. We believe this discrepancy is an artefact of the Hall effect measurements from which the concentrations were calculated. The authors assumed a Hall factor r_H of unity when extracting the carrier concentrations when in practice the Hall factor will depend on the band structure, temperature, and doping, and will likely be direction dependent. Although, the carrier concentration of a sample should be independent of the orientation of the sample, the measured carrier concentrations vary by up to three times suggesting that r_H actually deviates from unity significantly. This anomalous behaviour was also recently highlighted by Ma *et al.* [123] who calculated the mobility of SnSe using DFPT+Wannier as implemented in EPW. To compare directly to their EPW results, we have used the same carrier concentrations in our calculations. We further note that use of a constant Hall factor may also explain the large anisotropy of the carrier mobilities between the b and c directions [122] which is not reproduced in DFPT+Wannier calculations.

Supplementary Table 4. Materials parameters used to compute scatterings rates. **C** is the elastic tensor in Voigt notation, with the unit GPa. $\boldsymbol{\epsilon}_{\mathrm{s}}$ and $\boldsymbol{\epsilon}_{\infty}$ are the static and high-frequency dielectric constants in ϵ_0. $\mathbf{D}^{\mathrm{vb}}$ and $\mathbf{D}^{\mathrm{cb}}$ are the absolute deformation potentials at the valence and conduction band edge, respectively. d is the dimensionless piezoelectric coefficient. ω_{po} is the effective polar phonon frequency given in THz. For all tensor properties, components that are not explicitly listed are zero

Material	C_{11}	C_{22}	C_{33}	C_{44}	C_{55}	C_{66}	C_{12}	C_{13}	C_{23}	$\epsilon_{\mathrm{s},11}$	$\epsilon_{\mathrm{s},22}$	$\epsilon_{\mathrm{s},33}$	$\epsilon_{\infty,11}$	$\epsilon_{\infty,22}$	$\epsilon_{\infty,33}$	D_{11}^{vb}	D_{22}^{vb}	D_{33}^{vb}	D_{11}^{cb}	D_{22}^{cb}	D_{33}^{cb}	d	ω_{po}
GaN	325	325	358	90	90	107	112	78	78	5.9	5.9	6.1	10.5	10.5	11.7	8.2	8.2	8.5	15.0	15.0	15.2	0.0	19.0
GaP	125	125	125	65	65	65	52	52	52	10.6	10.6	10.6	12.9	12.9	12.9	0.4	0.4	0.4	13.0	13.0	13.0	0.0	10.3
GaAs	99	99	99	51	51	51	41	41	41	10.3	10.3	10.3	12.2	12.2	12.2	1.2	1.2	1.2	8.6	8.6	8.6	0.0	8.2
InP	87	87	87	42	42	42	46	46	46	13.2	13.2	13.2	16.5	16.5	16.5	1.6	1.6	1.6	5.7	5.7	5.7	0.0	10.3
ZnO	188	188	205	37	37	39	109	92	92	3.8	3.8	3.8	10.5	10.5	11.4	7.6	7.6	8.2	9.1	9.1	9.5	0.0	11.2
ZnS	96	96	96	46	46	46	55	55	55	5.9	5.9	5.9	9.4	9.4	9.4	0.5	0.5	0.5	7.0	7.0	7.0	0.1	7.9
ZnSe	82	82	82	37	37	37	47	47	47	7.3	7.3	7.3	10.7	10.7	10.7	0.8	0.8	0.8	5.7	5.7	5.7	0.0	5.9
CdS	80	80	85	15	15	17	45	37	37	6.0	6.0	6.1	9.8	9.8	10.4	0.3	0.3	0.8	2.4	2.4	3.2	0.2	6.5
CdSe	66	66	72	13	13	15	36	31	31	8.8	8.8	8.7	12.4	12.4	13.0	0.2	0.2	0.6	2.4	2.4	2.9	0.1	4.7
CdTe	47	47	47	19	19	19	30	30	30	9.3	9.3	9.3	12.8	12.8	12.8	0.1	0.1	0.1	2.9	2.9	2.9	0.0	3.9
SiC	382	382	382	241	241	241	126	126	126	7.0	7.0	7.0	10.3	10.3	10.3	5.6	5.6	5.6	3.4	3.4	3.4	0.2	23.2
PbS	121	121	121	20	20	20	18	18	18	15.6	15.6	15.6	277.7	277.7	277.7	1.6	1.6	1.6	1.0	1.0	1.0	0.0	6.6
PbTe	108	108	108	14	14	14	6	6	6	26.2	26.2	26.2	380.0	380.0	380.0	0.9	0.9	0.9	3.2	3.2	3.2	0.0	3.2
$MAPbI_3$ [a]	43	55	53	10	20	11	33	31	17	31.7	97.5	66.7	5.1	5.2	5.1	4.0	4.7	4.0	2.8	3.1	2.5	0.0	2.6
SnO_2	376	215	215	178	84	84	127	127	136	4.9	4.6	4.6	10.5	13.6	13.6	0.9	1.5	1.5	10.7	13.2	13.2	0.0	10.4
SnS	29	37	71	18	29	18	12	27	10	12.6	12.6	14.2	26.7	24.5	37.7	7.5	2.9	0.2	0.1	3.9	1.3	0.0	3.2
SnSe	30	39	67	12	28	14	13	28	8	16.9	15.3	18.7	32.3	27.1	46.3	13.8	15.9	14.7	11.2	9.8	14.5	0.0	3.2
Bi_2Te_3	55	55	10	8	8	20	14	8	8	35.0	35.0	27.7	117.2	117.2	35.2	1.1	1.8	1.8	3.2	3.8	3.8	0.0	3.4
BiCuOSe	131	131	83	29	29	40	50	47	47	16.1	16.7	13.7	122.4	122.4	43.4	2.1	2.1	2.9	5.3	5.3	1.2	0.0	3.4
$CuAlO_2$	294	294	509	39	39	102	90	103	103	6.1	6.1	4.6	10.8	10.8	7.3	2.5	2.5	4.8	7.1	7.1	10.2	0.0	14.0
Si	144	144	144	75	75	75	53	53	53	13.0	13.0	13.0	13.0	13.0	13.0	6.5	1.1	1.1	8.1	0.5	0.5	0.0	0.0
Ba_2BiAu	69	69	69	17	17	17	18	18	18	37.2	37.2	37.2	22.2	22.2	22.2	3.0	3.0	3.0	2.9	2.9	2.9	0.0	1.8
NbFeSb	309	309	309	67	67	67	95	95	95	44.7	44.7	44.7	24.7	24.7	24.7	1.1	1.1	1.1	0.6	1.6	1.6	0.0	7.4

[a] MA = CH_3NH_3

Supplementary Table 5. Band gaps and **k**-point meshes used to compute scatterings rates. ε_g^{HSE} and ε_g^{exp} are the band gaps calculated using the HSE06 functional and taken from experiment, respectively, with the references given in square brackets. The coarse **k**-point mesh of the electronic band structures computed using density functional theory (DFT) are compared to the dense mesh obtained through Fourier interpolation

			**k**-point mesh	
Material	ε_g^{HSE} (eV)	ε_g^{exp} (eV)	DFT	Interpolated
GaN	3.06 [70]	3.26 [71]	20 × 20 × 12	183 × 183 × 97
GaP	2.37[a]	2.24 [72]	18 × 18 × 18	105 × 105 × 105
GaAs	1.33 [73]	1.52 [71]	17 × 17 × 17	143 × 143 × 143
InP	1.48 [74]	1.42 [71]	16 × 16 × 16	151 × 151 × 151
ZnO	2.55 [75]	3.37 [76]	20 × 20 × 12	145 × 145 × 77
ZnS	3.22[a]	3.72 [77]	18 × 18 × 18	133 × 133 × 133
ZnSe	2.24[a]	2.82 [78]	17 × 17 × 17	99 × 99 × 99
CdS	2.12[a]	2.48 [79]	15 × 15 × 9	87 × 87 × 47
CdSe	1.46[a]	1.73 [80]	15 × 15 × 9	87 × 87 × 47
CdTe	1.34[a]	1.48 [81]	15 × 15 × 15	89 × 89 × 89
SiC	2.35[a]	2.36 [82]	22 × 22 × 22	125 × 125 × 125
PbS	0.84 [83]	0.37 [76]	16 × 16 × 16	119 × 119 × 119
PbTe	1.45[a]	0.31 [84]	15 × 15 × 15	101 × 101 × 101
$MAPbI_3$[b]	2.43[a]	1.63 [85]	7 × 4 × 6	51 × 33 × 47
SnO_2	2.88 [86]	3.60 [87]	19 × 13 × 13	135 × 91 × 91
SnS	1.58[a]	1.42 [88]	15 × 14 × 5	57 × 51 × 19
SnSe	1.10 [89]	0.90 [90]	13 × 13 × 5	51 × 49 × 17
Bi_2Te_3	0.93[a]	0.13 [91]	11 × 11 × 11	61 × 61 × 61
BiCuOSe	1.08[a]	0.80 [92]	15 × 15 × 11	47 × 47 × 21
$CuAlO_2$	3.52 [93]	2.97 [94]	14 × 14 × 4	57 × 57 × 13
Si	1.15 [75]	1.14 [76]	18 × 18 × 18	105 × 105 × 105
Ba_2BiAu	0.88[a]	—	11 × 11 × 11	41 × 41 × 41
NbFeSb	1.26[a]	0.51 [95]	16 × 16 × 16	45 × 45 × 45

[a] This work.
[b] MA = CH_3NH_3

Supplementary Table 6. Summary of temperature and doping conditions used for computing electron mobility. References provided to DFPT+Wannier calculations and experimental measurements performed at the same doping and temperature conditions, which are used in the comparison of electron mobilities in the main text and Supplemental Material. For anisotropic materials that were measured as single crystals, we also report the axis along which transport properties were measured and calculated

Material	Doping	T (K)	n (cm^{-3})	Exp.	DFPT+Wannier	Axis
GaN	n-type	150–500	3.0×10^{16}–5.5×10^{16}	[96]	[97]	—
GaP	n-type	100–500	3.0×10^{16}	[98]	—	—
GaAs	n-type	200–1000	3.0×10^{13}	[18]	[99]	—
GaAs	p-type	300	3.0×10^{13}–8.6×10^{19}	[100, 101]	—	—
InP	n-type	150–700	1.5×10^{16}	[102]	—	—
ZnO	n-type	300–1000	8.2×10^{16}	[103]	—	—
ZnS	n-type	300–650	1.0×10^{16}	[104]	—	—
ZnSe	n-type	200–1300	4.0×10^{14}–2.0×10^{15}	[105, 106]	—	—
CdS	n-type	100–400	5.0×10^{15}	[107]	—	—
CdSe	n-type	150–1300	1.0×10^{16}–1.0×10^{18}	[108, 109]	—	—
CdTe	n-type	100–1200	5.4×10^{14}–1.4×10^{17}	[110, 111]	—	—
CdTe	p-type	550–1000	1.4×10^{16}–6.7×10^{16}	[111]	—	—
SiC	n-type	100–850	3.7×10^{15}–2.5×10^{16}	[112]	[113]	—
PbS	n-type	300–750	3.6×10^{17}	[114]	—	—
PbTe	n-type	100–300	1.1×10^{18}	[115]	[116]	—
$MAPbI_3$ [a]	n-type	100–350	1.0×10^{14}	[117, 118]	[119]	—
SnO_2	n-type	300–700	1.0×10^{17}	[120]	—	—
SnS	p-type	100–600	4.3×10^{17}–5.6×10^{17}	[121]	—	a
SnSe	p-type	300–600	3.0×10^{17}	[122]	[123]	b
Bi_2Te_3	n-type	100–300	3.3×10^{19}	[124]	—	b–c
Bi_2Te_3	p-type	100–250	3.0×10^{18}	[125]	—	b–c
BiCuOSe	p-type	300–650	2.2×10^{18}–6.4×10^{19}	[126]	—	—
$CuAlO_2$	p-type	300–430	1.3×10^{17}–7.4×10^{18}	[94]	—	a–b
Si	n-type	300	2.0×10^{14}–4.4×10^{18}	[127]	[3]	—
Ba_2BiAu	n-type	300	1×10^{14}	[43]	—	—
NbFeSb	p-type	300	2×10^{20}	[44, 45]	—	—

[a] MA = CH_3NH_3

Supplementary Table 7. Summary of temperature and doping conditions used for computing Seebeck coefficient. References provided to experimental measurements performed at the same doping and temperature conditions, which are used in the comparison of Seebeck coefficients in the main text and Supplemental Material

Material	Doping	T (K)	n (cm^{-3})	Exp.
GaN	n-type	100–300	1.3×10^{19}	[128]
GaAs	n-type	400–750	3.5×10^{17}	[129]
GaAs	p-type	350–750	6.4×10^{19}	[130]
InP	n-type	150–700	2.1×10^{17}	[131]
ZnO	n-type	200–1000	5.2×10^{17}	[132]
CdS	n-type	130–300	2.8×10^{15}	[133]
PbS	n-type	300–800	2.5×10^{19}	[134]
SnO_2	n-type	300–800	8.2×10^{18}	[135]
SnSe	p-type	300–600	3.0×10^{17}	[122]
Si	n-type	300	1×10^{14}–1×10^{19}	[136, 137]

II. SUPPLEMENTARY DISCUSSION

A. Mobility results

1. *Temperature and carrier dependent mobility*

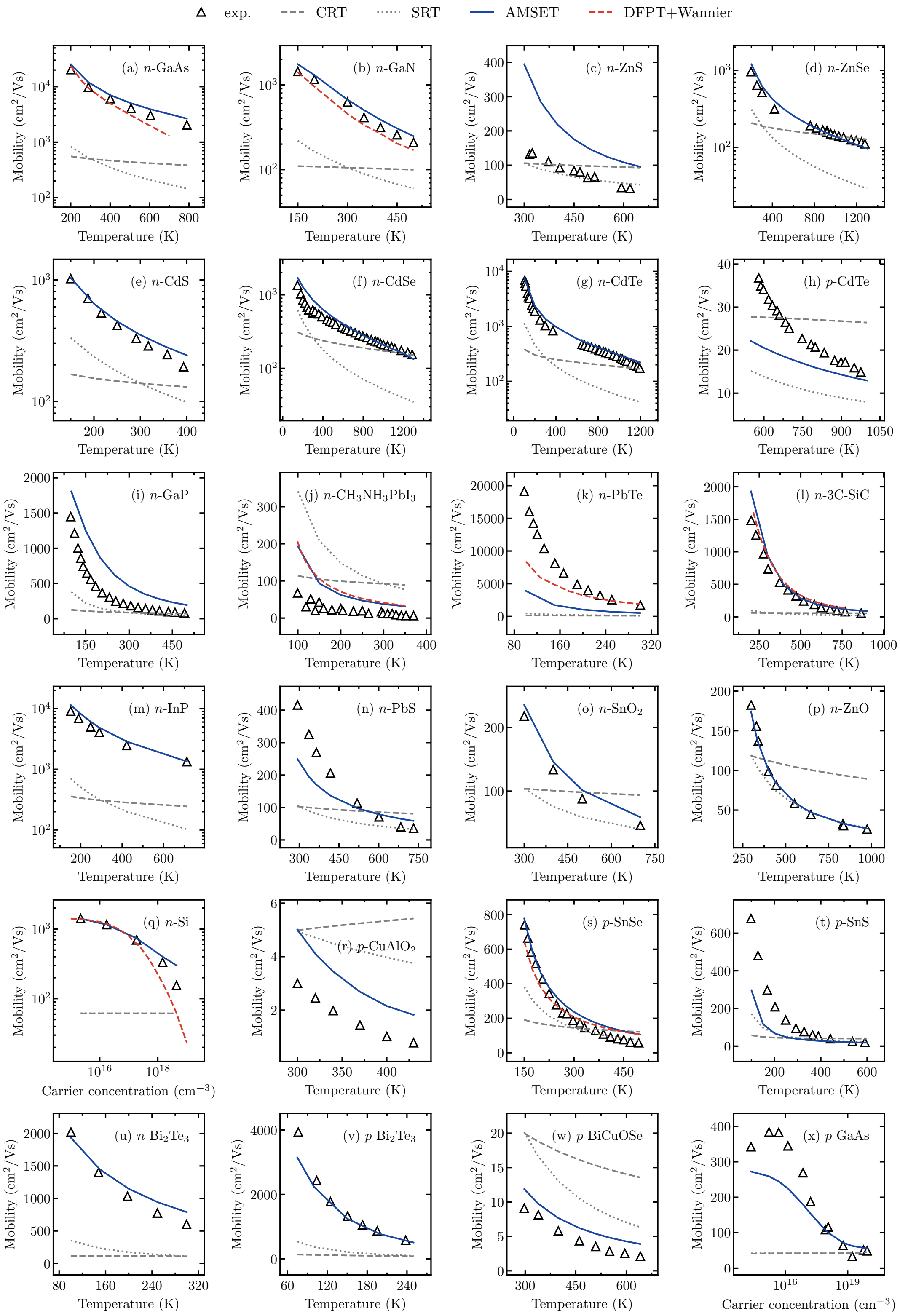

Supplementary Figure 8. Mobility against temperature or carrier-concentration for all test materials, computed using the HSE06 band gap. Constant relaxation time (CRT) results performed using $\tau = 10\,\text{fs}$. Scaled relaxation time (SRT) performed using $\tau_T = 10 \times \frac{300}{T}$ fs. For anisotropic materials such as SnSe and Bi_2Te_3, the calculated and experimental results are reported along particular directions as reported in Supplementary Table 6.

2. *Scattering limited mobilities*

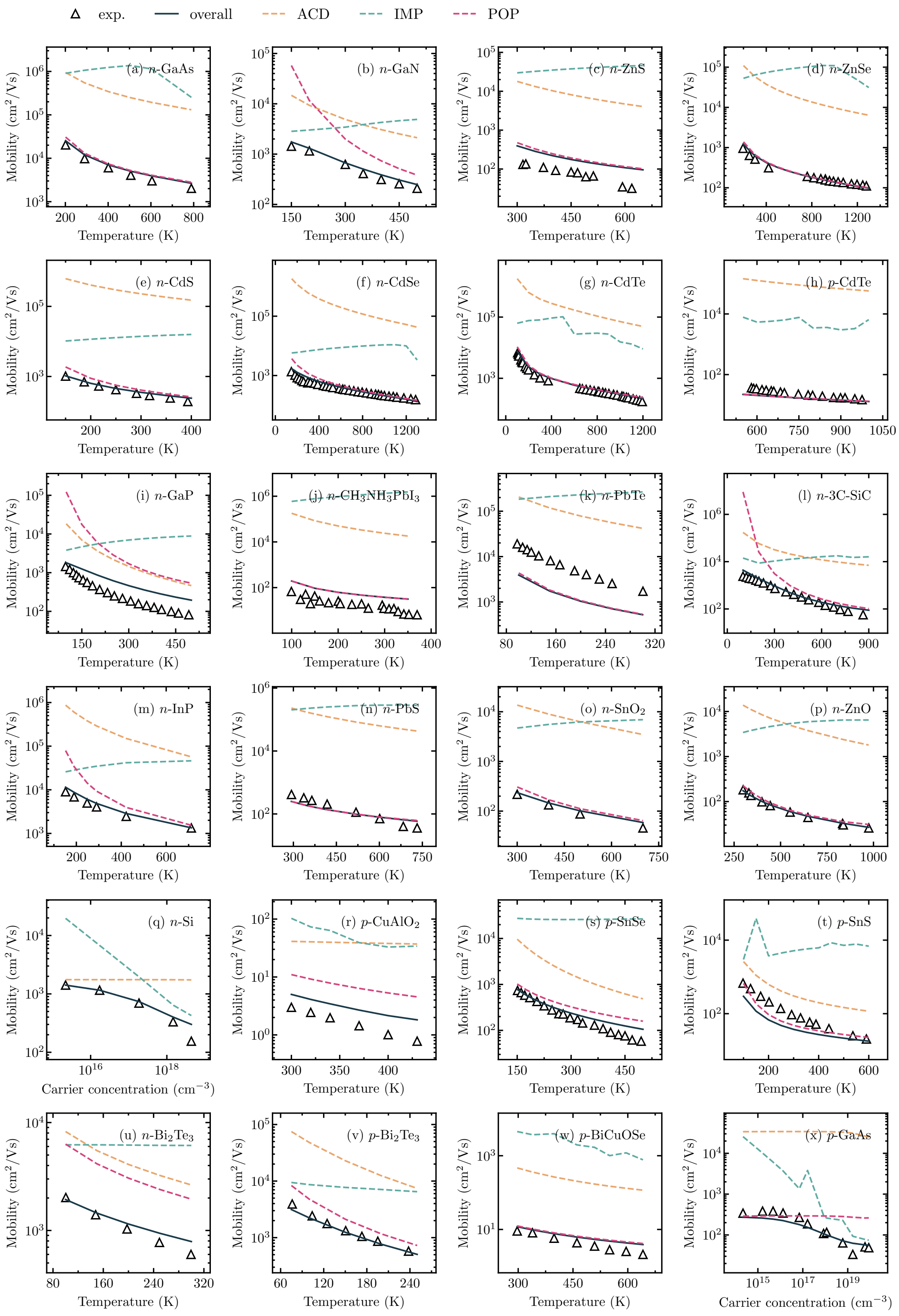

Supplementary Figure 9. Mobility limited by different scattering mechanisms against temperature or carrier-concentration for all test materials, computed using the HSE06 band gap. For anisotropic materials such as SnSe and Bi_2Te_3, the calculated and experimental results are reported along particular directions as reported in Supplementary Table 6.

3. Mobility calculated using the HSE06 functional

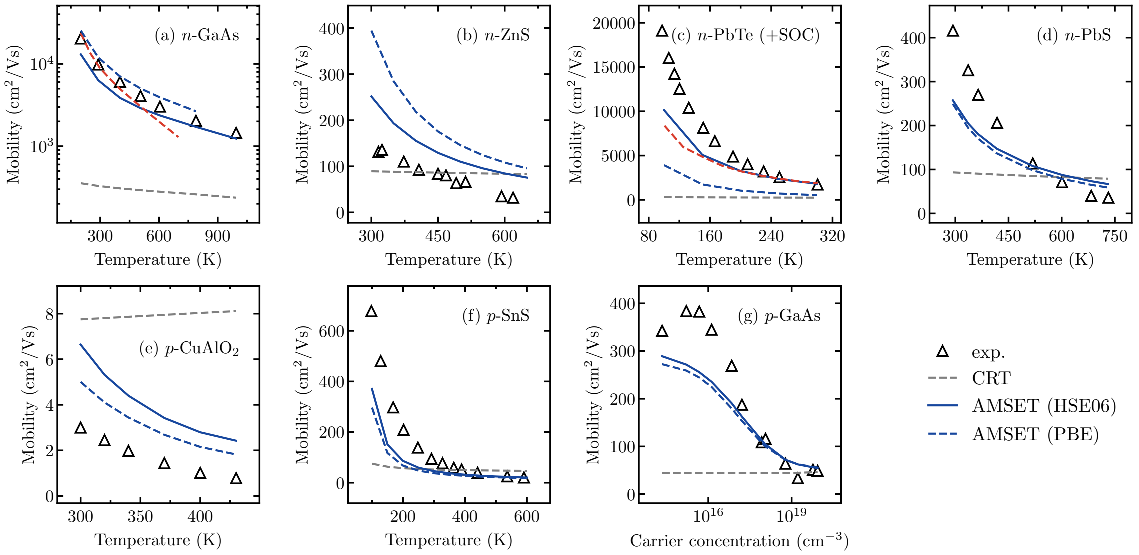

Supplementary Figure 10. Mobility against temperature or carrier-concentration for a set of test materials, computed using HSE06 electronic structures. PbTe calculations also include spin–orbit coupling effects. For anisotropic materials such as SnS and $CuAlO_2$, the calculated and experimental results are reported along particular directions as reported in Supplementary Table 6.

B. Seebeck coefficient results

1. *Temperature- and carrier concentration-dependent Seebeck coefficient*

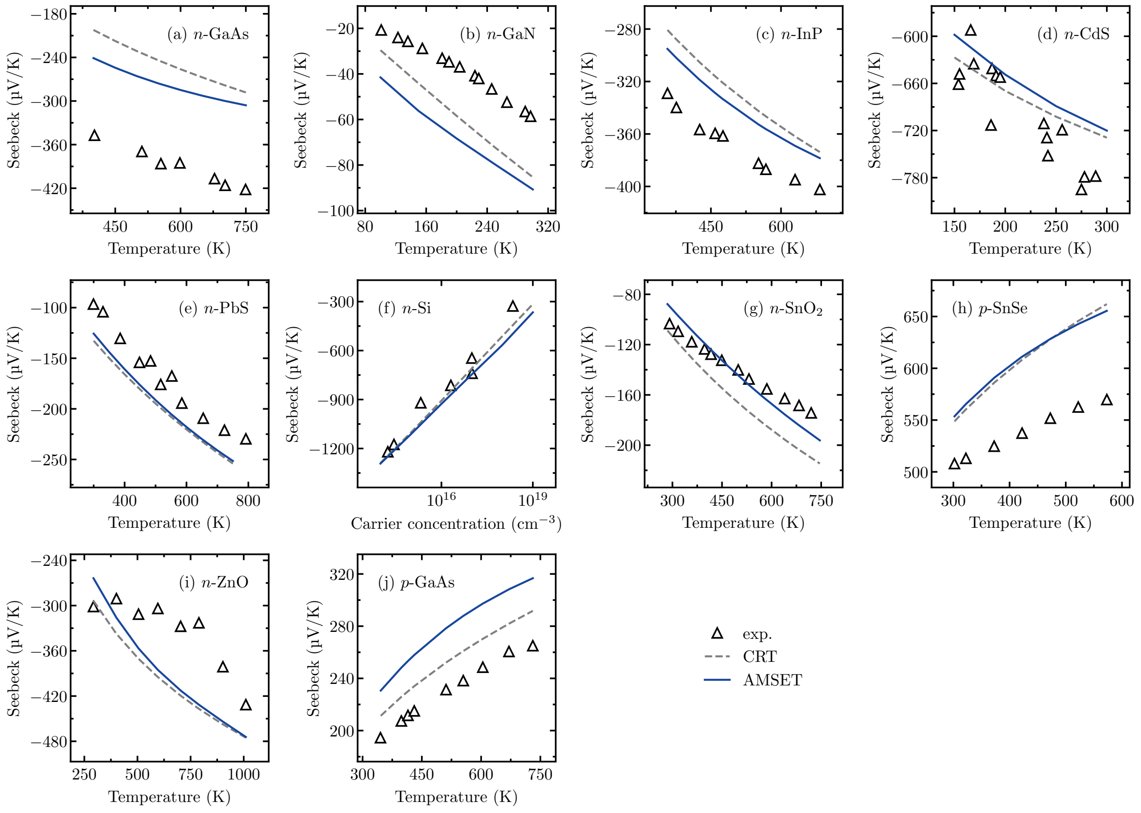

Supplementary Figure 11. Seebeck coefficient against temperature for all test materials, computed using the HSE06 band gap.

2. *Seebeck coefficient calculated using the HSE06 functional*

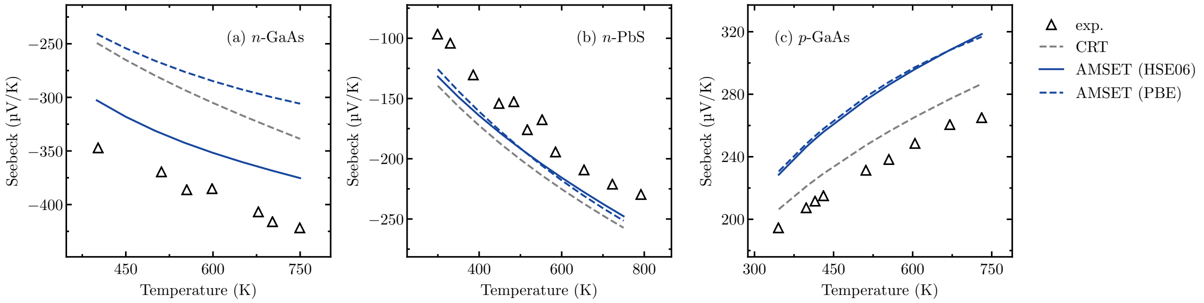

Supplementary Figure 12. Seebeck coefficient against temperature for a set of test materials computed using HSE06 electronic structures.

C. Scattering rate comparison

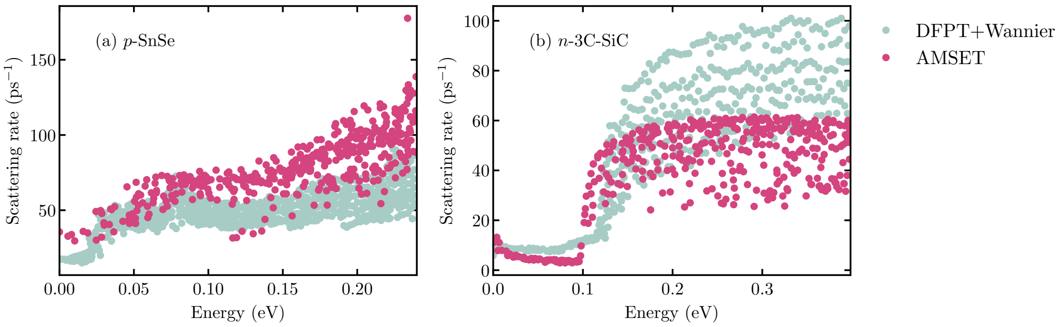

Supplementary Figure 13. Computed scattering rates compared against DFPT+Wannier calculations [3, 99, 113, 123]. Results calculated at 300 K using the the lowest carrier concentrations for each material given in Supplementary Table. 6

.

D. Comparison against CRT and DFPT+Wannier

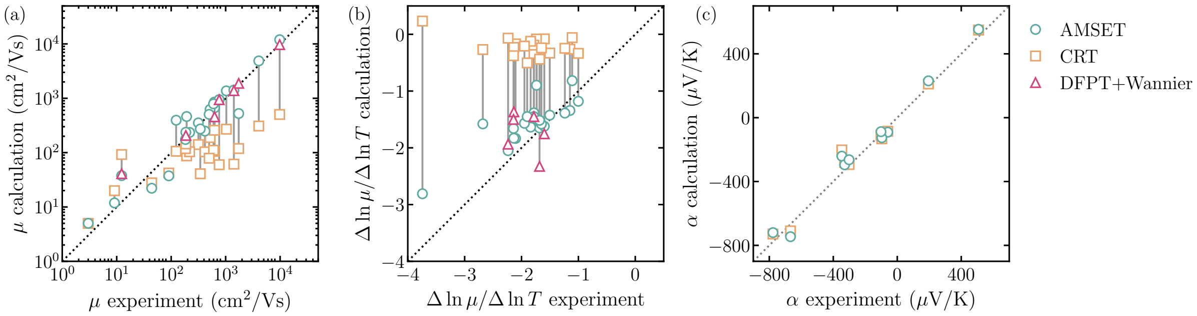

Supplementary Figure 14. Comparison between AMSET, constant relaxation time approximation calculations, DFPT+Wannier calculations, and experiments for (a) carrier mobilities at 300 K (b) the exponential temperature trend of carrier mobilities, and (c) Seebeck coefficients at 300 K.

E. Band structures

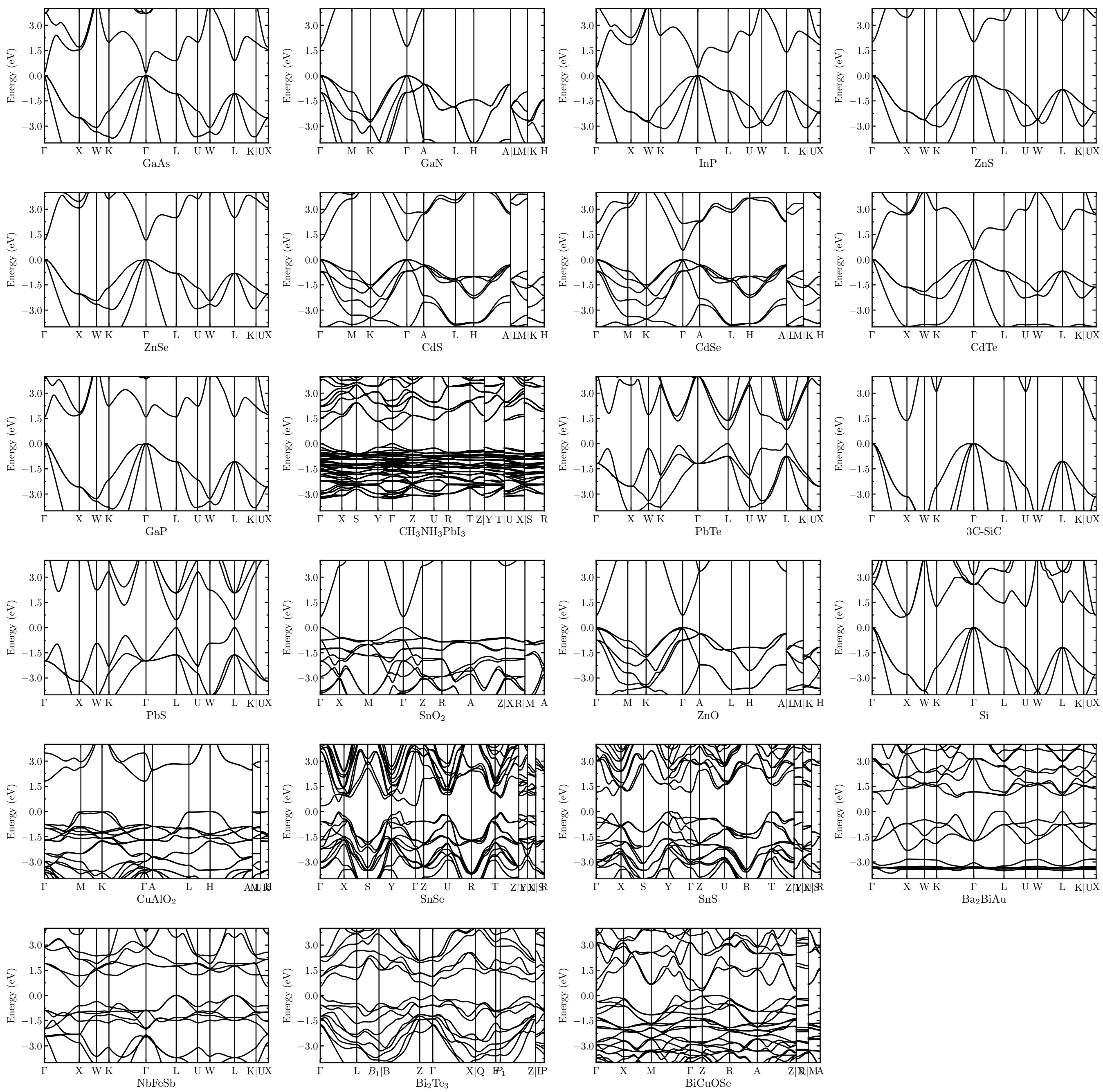

Supplementary Figure 15. Band structures (pre-scissor operation) calculated using the PBE exchange–correlation functional, interpolated from a uniform **k**-point mesh using the BOLTZTRAP2 package.

[1] J. Ziman, Electrons and Phonons, Oxford University Press (1960).
[2] S. Poncé, W. Li, S. Reichardt, and F. Giustino, First-principles calculations of charge carrier mobility and conductivity in bulk semiconductors and two-dimensional materials, Rep. Prog. Phys. **83**, 036501 (2020).
[3] S. Poncé, E. R. Margine, and F. Giustino, Towards predictive many-body calculations of phonon-limited carrier mobilities in semiconductors, Phys. Rev. B **97**, 121201 (2018).
[4] W. Li, Electrical transport limited by electron-phonon coupling from Boltzmann transport equation: An *ab initio* study of Si, Al, and MoS 2, Phys. Rev. B **92**, 075405 (2015).
[5] G. Grimvall, *The Electron-Phonon interaction in metals* (North-Holland, Amsterdam, 1981).
[6] F. Giustino, M. L. Cohen, and S. G. Louie, Electron-phonon interaction using Wannier functions, Phys. Rev. B **76**, 165108 (2007).
[7] H. Brooks, Scattering by ionized impurities in semiconductors, in *Phys. Rev.*, Vol. 83 (1951) pp. 879–879.
[8] C. Herring and E. Vogt, Transport and deformation-potential theory for many-valley semiconductors with anisotropic scattering, Phys. Rev. **101**, 944 (1956).
[9] J. Bardeen and W. Shockley, Deformation potentials and mobilities in non-polar crystals, Phys. Rev. **80**, 72 (1950).
[10] F. S. Khan and P. B. Allen, Deformation potentials and electron-phonon scattering: Two new theorems, Phys. Rev. B **29**, 3341 (1984).
[11] E. Kartheuser and S. Rodriguez, Deformation potentials and the electron-phonon interaction in metals, Phys. Rev. B **33**, 772 (1986).
[12] R. Resta, Deformation-potential theorem in metals and in dielectrics, Phys. Rev. B **44**, 11035 (1991).
[13] H. Meijer and D. Polder, Note on polar scattering of conduction electrons in regular crystals, Physica **19**, 255 (1953).
[14] W. A. Harrison, Mobility in zinc blende and indium antimonide, Phys. Rev. **101**, 903 (1956).
[15] D. Rode, Low-field electron transport, in *Semiconductors and semimetals*, Vol. 10 (Elsevier, 1975) pp. 1–89.
[16] H. Fröhlich, Electrons in lattice fields, Adv. Phys. **3**, 325 (1954).
[17] M. Born, Quantenmechanik der stoßvorgänge, Z. Phys. **38**, 803 (1926).
[18] D. Rode and S. Knight, Electron transport in GaAs, Phys. Rev. B **3**, 2534 (1971).
[19] L. Onsager, Reciprocal relations in irreversible processes. i., Phys. Rev. **37**, 405 (1931).
[20] G. K. Madsen, J. Carrete, and M. J. Verstraete, BoltzTraP2, a program for interpolating band structures and calculating semi-classical transport coefficients, Comput. Phys. Commun. **231**, 140 (2018).
[21] P. E. Blöchl, O. Jepsen, and O. K. Andersen, Improved tetrahedron method for Brillouin-zone integrations, Phys. Rev. B **49**, 16223 (1994).
[22] G. K. Madsen, J. Carrete, and M. J. Verstraete, BoltzTraP2, a program for interpolating band structures and calculating semi-classical transport coefficients, Comput. Phys. Commun. **231**, 140 (2018).
[23] G. K. Madsen and D. J. Singh, BoltzTraP. A code for calculating band-structure dependent quantities, Comput. Phys. Commun. **175**, 67 (2006).
[24] R. N. Euwema, D. J. Stukel, T. C. Collins, J. S. Dewitt, and D. G. Shankland, Crystalline Interpolation with Applications to Brillouin-Zone Averages and Energy-Band Interpolation, Phys. Rev. **178**, 1419 (1969).
[25] D. Koelling and J. Wood, On the interpolation of eigenvalues and a resultant integration scheme, J. Comput. Phys. **67**, 253 (1986).
[26] D. G. Shankland, Fourier transformation by smooth interpolation, Int. J. Quantum Chem. **5**, 497 (2009).
[27] W. E. Pickett, H. Krakauer, and P. B. Allen, Smooth Fourier interpolation of periodic functions, Phys. Rev. B **38**, 2721 (1988).
[28] G. Lehmann and M. Taut, On the numerical calculation of the density of states and related properties, Phys. Status Solidi B **54**, 469 (1972).
[29] H. Xiao and Z. Gimbutas, A numerical algorithm for the construction of efficient quadrature rules in two and higher dimensions, Comput. Math. with Appl. **59**, 663 (2010).
[30] M. Festa and A. Sommariva, Computing almost minimal formulas on the square, J. Comput. Appl. Math **236**, 4296 (2012).
[31] N. Schlömer, N. R. Papior, R. Zetter, M. Ancellin, and D. Arnold, nschloe/quadpy v0.14.11 (2020).
[32] C. Friedrich, Tetrahedron integration method for strongly varying functions: Application to the G T self-energy, Phys. Rev. B **100**, 075142 (2019).
[33] A. Ganose, AMSET: *ab initio* scattering and transport (2020).
[34] S. P. Ong, W. D. Richards, A. Jain, G. Hautier, M. Kocher, S. Cholia, D. Gunter, V. L. Chevrier, K. A. Persson, and G. Ceder, Python Materials Genomics (pymatgen): A robust, open-source python library for materials analysis, Comput. Mater. Sci. **68**, 314 (2013).
[35] G. K. Madsen and D. J. Singh, BoltzTraP. a code for calculating band-structure dependent quantities, Comput. Phys. Commun. **175**, 67 (2006).
[36] A. Togo and I. Tanaka, `Spglib`: A software library for crystal symmetry search, ArXiv180801590 Cond-Mat (2018), arXiv:1808.01590 [cond-mat].
[37] J. D. Hunter, Matplotlib: A 2D Graphics Environment, Comput. Sci. Eng. **9**, 90 (2007).
[38] S. Van Der Walt, S. C. Colbert, and G. Varoquaux, The NumPy array: A structure for efficient numerical computation, Comput. Sci. Eng. **13**, 22 (2011), arXiv:1102.1523.
[39] SciPy 1.0 Contributors, P. Virtanen, R. Gommers, T. E. Oliphant, M. Haberland, T. Reddy, D. Cournapeau, E. Burovski, P. Peterson, W. Weckesser, J. Bright, S. J. van der Walt, M. Brett, J. Wilson, K. J. Millman, N. Mayorov, A. R. J. Nelson, E. Jones, R. Kern, E. Larson, C. J. Carey, İ. Polat, Y. Feng, E. W. Moore, J. VanderPlas, D. Laxalde, J. Perktold, R. Cimrman, I. Henriksen, E. A. Quintero, C. R. Harris, A. M. Archibald, A. H. Ribeiro, F. Pedregosa, and P. van Mulbregt, SciPy 1.0: Fundamental algorithms for scientific computing in Python, Nat. Methods **17**, 261 (2020).

[40] K. Bystrom, D. Broberg, S. Dwaraknath, K. A. Persson, and M. Asta, Pawpyseed: Perturbation-extrapolation band shifting corrections for point defect calculations, ArXiv190411572 Cond-Mat (2019), arXiv:1904.11572 [cond-mat].

[41] M. Folk, G. Heber, Q. Koziol, E. Pourmal, and D. Robinson, An overview of the HDF5 technology suite and its applications, in *Proceedings of the EDBT/ICDT 2011 Workshop on Array Databases* (2011) pp. 36–47.

[42] A. M. Ganose, A. J. Jackson, and D. O. Scanlon, sumo: Command-line tools for plotting and analysis of periodic *ab initio* calculations, Journal of Open Source Software **3**, 717 (2018).

[43] J. Park, Y. Xia, and V. Ozoliņš, High Thermoelectric Power Factor and Efficiency from a Highly Dispersive Band in Ba_2BiAu, Phys. Rev. Applied **11**, 014058 (2019).

[44] J. Zhou, H. Zhu, T.-H. Liu, Q. Song, R. He, J. Mao, Z. Liu, W. Ren, B. Liao, D. J. Singh, Z. Ren, and G. Chen, Large thermoelectric power factor from crystal symmetry-protected non-bonding orbital in half-Heuslers, Nat. Commun. **9**, 1721 (2018).

[45] G. Samsonidze and B. Kozinsky, Accelerated screening of thermoelectric materials by first-principles computations of electron–phonon scattering, Adv. Energy Mater. **8**, 1800246 (2018).

[46] D. Chattopadhyay and H. J. Queisser, Electron scattering by ionized impurities in semiconductors, Rev. Mod. Phys. **53**, 745 (1981).

[47] G. Dresselhaus, A. F. Kip, and C. Kittel, Cyclotron Resonance of Electrons and Holes in Silicon and Germanium Crystals, Phys. Rev. **98**, 368 (1955).

[48] P. Y. Yu and M. Cardona, *Fundamentals of semiconductors*, edited by H. E. Stanley and W. T. Rhodes (Springer, 2010).

[49] P. Norton, T. Braggins, and H. Levinstein, Impurity and Lattice Scattering Parameters as Determined from Hall and Mobility Analysis in n-Type Silicon, Phys. Rev. B **8**, 5632 (1973).

[50] C.-H. Park, F. Giustino, J. L. McChesney, A. Bostwick, T. Ohta, E. Rotenberg, M. L. Cohen, and S. G. Louie, Van Hove singularity and apparent anisotropy in the electron-phonon interaction in graphene, Phys. Rev. B **77**, 113410 (2008).

[51] P. Hohenberg and W. Kohn, Inhomogeneous Electron Gas, Phys. Rev. **136**, B864 (1964).

[52] W. Kohn and L. J. Sham, Self-Consistent Equations Including Exchange and Correlation Effects, Phys. Rev. **140**, A1133 (1965).

[53] G. Kresse and J. Hafner, Ab initio molecular-dynamics simulation of the liquid-metal–amorphous-semiconductor transition in germanium, Phys. Rev. B **49**, 14251 (1994).

[54] G. Kresse and J. Furthmüller, Efficient iterative schemes for ab initio total-energy calculations using a plane-wave basis set, Phys. Rev. B **54**, 11169 (1996).

[55] G. Kresse and J. Furthmüller, Efficiency of ab-initio total energy calculations for metals and semiconductors using a plane-wave basis set, Comput. Mater. Sci. **6**, 15 (1996).

[56] J. P. Perdew and W. Yue, Accurate and simple density functional for the electronic exchange energy: Generalized gradient approximation, Phys. Rev. B **33**, 8800 (1986).

[57] J. P. Perdew, K. Burke, and M. Ernzerhof, Generalized gradient approximation made simple, Phys. Rev. Lett. **77**, 3865 (1996).

[58] P. E. Blöchl, Projector augmented-wave method, Phys. Rev. B **50**, 17953 (1994), arXiv:1408.4701v2.

[59] G. Kresse and D. Joubert, From ultrasoft pseudopotentials to the projector augmented-wave method, Phys. Rev. B **59**, 1758 (1999).

[60] K. Mathew, J. H. Montoya, A. Faghaninia, S. Dwarakanath, M. Aykol, H. Tang, I. heng Chu, T. Smidt, B. Bocklund, M. Horton, J. Dagdelen, B. Wood, Z. K. Liu, J. Neaton, S. P. Ong, K. Persson, and A. Jain, Atomate: A high-level interface to generate, execute, and analyze computational materials science workflows, Comput. Mater. Sci. **139**, 140 (2017).

[61] Atomate v0.8.3 (2018).

[62] S. Baroni and R. Resta, *Ab Initio* calculation of the macroscopic dielectric constant in silicon, Phys. Rev. B **33**, 7017 (1986).

[63] M. Gajdoš, K. Hummer, G. Kresse, J. Furthmüller, and F. Bechstedt, Linear optical properties in the projector-augmented wave methodology, Phys. Rev. B **73**, 045112 (2006), arXiv:cond-mat/0510491.

[64] M. de Jong, W. Chen, T. Angsten, A. Jain, R. Notestine, A. Gamst, M. Sluiter, C. Krishna Ande, S. van der Zwaag, J. J. Plata, C. Toher, S. Curtarolo, G. Ceder, K. A. Persson, and M. Asta, Charting the complete elastic properties of inorganic crystalline compounds, Sci. Data **2**, 150009 (2015).

[65] S.-H. Wei and A. Zunger, Predicted band-gap pressure coefficients of all diamond and zinc-blende semiconductors: Chemical trends, Phys. Rev. B **60**, 5404 (1999).

[66] R. Resta, L. Colombo, and S. Baroni, Absolute deformation potentials in semiconductors, Phys. Rev. B **41**, 12358 (1990).

[67] Y.-H. Li, A. Walsh, S. Chen, W.-J. Yin, J.-H. Yang, J. Li, J. L. F. Da Silva, X. G. Gong, and S.-H. Wei, Revised *ab initio* natural band offsets of all group IV, II-VI, and III-V semiconductors, Appl. Phys. Lett. **94**, 212109 (2009).

[68] C. Verdi and F. Giustino, Fröhlich electron-phonon vertex from first principles, Phys. Rev. Lett. **115**, 176401 (2015).

[69] J. Sjakste, N. Vast, M. Calandra, and F. Mauri, Wannier interpolation of the electron-phonon matrix elements in polar semiconductors: Polar-optical coupling in GaAs, Phys. Rev. B **92**, 054307 (2015).

[70] A. Stroppa and G. Kresse, Unraveling the Jahn-Teller effect in Mn-doped GaN using the Heyd-Scuseria-Ernzerhof hybrid functional, Phys. Rev. B **79**, 201201 (2009).

[71] I. Vurgaftman, J. R. Meyer, and L. R. Ram-Mohan, Band parameters for III–V compound semiconductors and their alloys, J. Appl. Phys. **89**, 5815 (2001).

[72] L. M. Foster and M. Pilkuhn, Electroluminescence near band gap in gallium phosphide containing shallow donor and acceptor levels, Appl. Phys. Lett. **7**, 65 (1965).

[73] Y.-S. Kim, M. Marsman, G. Kresse, F. Tran, and P. Blaha, Towards efficient band structure and effective mass calculations for III-V direct band-gap semiconductors, Phys. Rev. B **82**, 205212 (2010).

[74] Y.-S. Kim, K. Hummer, and G. Kresse, Accurate band structures and effective masses for InP, InAs, and InSb

using hybrid functionals, Phys. Rev. B **80**, 035203 (2009).

[75] Y. Hinuma, A. Grüneis, G. Kresse, and F. Oba, Band alignment of semiconductors from density-functional theory and many-body perturbation theory, Phys. Rev. B **90**, 155405 (2014).

[76] O. Madelung, *Semiconductors: data handbook* (Springer Science & Business Media, 2012).

[77] T. K. Tran, W. Park, W. Tong, M. M. Kyi, B. K. Wagner, and C. J. Summers, Photoluminescence properties of ZnS epilayers, J. Appl. Phys. **81**, 2803 (1997).

[78] A. Mang, K. Reimann, and S. Rübenacke, Two-photon spectroscopy in ZnSe under hydrostatic pressure, in *Proceedings of the 22nd International Conference on the Physics of Semiconductors*, edited by D. J. Lockworth (World Scientific, Singapore, 1994) pp. 317–320.

[79] S. Ninomiya and S. Adachi, Optical properties of wurtzite CdS, J. Appl. Phys. **78**, 1183 (1995).

[80] S. Ninomiya and S. Adachi, Optical properties of cubic and hexagonal CdSe, J. Appl. Phys. **78**, 4681 (1995).

[81] P. Lemasson, Free excitons at room temperature in cadmium telluride: A photoelectrochemical evidence, Solid State Commun. **43**, 627 (1982).

[82] X. Liu, L. Li, Q. Li, Y. Li, and F. Lu, Optical and mechanical properties of C, Si, Ge, and 3C–SiC determined by first-principles theory using Heyd–Scuseria–Ernzerhof functional, Mater. Sci. Semicond. Process **16**, 1369 (2013).

[83] A. Walsh, Effects of reduced dimensionality on the electronic structure and defect chemistry of semiconducting hybrid organic–inorganic PbS solids, Proc. R. Soc. A **467**, 1970 (2011).

[84] H. Preier, Recent advances in lead-chalcogenide diode lasers, Appl. Phys. **20**, 189 (1979).

[85] M. E. Ziffer, J. C. Mohammed, and D. S. Ginger, Electroabsorption spectroscopy measurements of the exciton binding energy, electron–hole reduced effective mass, and band gap in the perovskite $CH_3NH_3PbI_3$, ACS Photonics **3**, 1060 (2016).

[86] F. Tran and P. Blaha, Importance of the Kinetic Energy Density for Band Gap Calculations in Solids with Density Functional Theory, J. Phys. Chem. A **121**, 3318 (2017).

[87] M. Batzill and U. Diebold, The surface and materials science of tin oxide, Prog. Surf. Sci. **79**, 47 (2005).

[88] A. Lambros, D. Geraleas, and N. Economou, Optical absorption edge in SnS, J. Phys. Chem. Solids **35**, 537 (1974).

[89] Y. Huang, C. Wang, X. Chen, D. Zhou, J. Du, S. Wang, and L. Ning, First-principles study on intrinsic defects of SnSe, RSC Adv. **7**, 27612 (2017).

[90] H. Soliman, D. Abdel Hady, K. Abdel Rahman, S. Youssef, and A. El-Shazly, Optical properties of tin-selenid films, Physica A **216**, 77 (1995).

[91] R. Sehr and L. Testardi, The optical properties of p-type Bi_2Te_3–Sb_2Te_3/sub> alloys between 2–15 microns, J. Phys. Chem. Solids **23**, 1219 (1962).

[92] Y. Liu, J. Lan, W. Xu, Y. Liu, Y.-L. Pei, B. Cheng, D.-B. Liu, Y.-H. Lin, and L.-D. Zhao, Enhanced thermoelectric performance of a BiCuSeO system via band gap tuning, Chem. Commun. **49**, 8075 (2013).

[93] D. O. Scanlon and G. W. Watson, Conductivity Limits in $CuAlO_2$ from Screened-Hybrid Density Functional Theory, J. Phys. Chem. Lett. **1**, 3195 (2010).

[94] J. Tate, H. L. Ju, J. C. Moon, A. Zakutayev, A. P. Richard, J. Russell, and D. H. McIntyre, Origin of p-type conduction in single-crystal $CuAlO_2$, Phys. Rev. B **80**, 165206 (2009).

[95] R. He, D. Kraemer, J. Mao, L. Zeng, Q. Jie, Y. Lan, C. Li, J. Shuai, H. S. Kim, Y. Liu, D. Broido, C.-W. Chu, G. Chen, and Z. Ren, Achieving high power factor and output power density in p-type half-Heuslers $Nb_{1-x}Ti_xFeSb$, Proc. Natl. Acad. Sci. **113**, 13576 (2016).

[96] D. Steigerwald, S. Rudaz, H. Liu, R. S. Kern, W. Götz, and R. Fletcher, III–V Nitride semiconductors for high-performance blue and green light-emitting devices, JOM **49**, 18 (1997).

[97] S. Poncé, D. Jena, and F. Giustino, Hole mobility of strained GaN from first principles, Phys. Rev. B **100**, 085204 (2019).

[98] R. C. Taylor, J. F. Woods, and M. R. Lorenz, Electrical and Optical Properties of Vapor-Grown GaP, J. Appl. Phys. **39**, 5404 (1968).

[99] J.-J. Zhou and M. Bernardi, *Ab Initio* electron mobility and polar phonon scattering in GaAs, Phys. Rev. B **94**, 201201 (2016).

[100] L. Janšák and T. S. Lagunova, Effect of magnetic field on the impurity conduction in p-GaAs, Phys. Stat. Sol. (a) **13**, K151 (1972).

[101] D. E. Hill, Activation Energy of Holes in Zn-Doped GaAs, J. Appl. Phys. **41**, 1815 (1970).

[102] V. V. Galavanov and N. V. Siukaev, On Mechanism of Electron Scattering in InP, phys. stat. sol. (b) **38**, 523 (1970).

[103] A. R. Hutson, Hall Effect Studies of Doped Zinc Oxide Single Crystals, Phys. Rev. **108**, 222 (1957).

[104] F. Kröger, Some optical and electrical measurements on blue fluorescent ZnS-Cl single crystals, Physica **22**, 637 (1956).

[105] M. Aven, High Electron Mobility in Zinc Selenide Through Low-Temperature Annealing, J. Appl. Phys. **42**, 1204 (1971).

[106] F. Smith, Evidence for a native donor in ZnSe from high temperature electrical measurements, Solid State Commun. **7**, 1757 (1969).

[107] B. Pödör, J. Balázs, and M. Hársy, Electron concentration and mobility in CdS single crystals, phys. stat. sol. (a) **8**, 613 (1971).

[108] R. A. Btirmeister and D. A. Stevenson, Electrical Properties of n-Type CdSe, phys. stat. sol. (b) **24**, 683 (1967).

[109] F. Smith, High temperature electrical properties of CdSe: Evidence for a native donor, Solid State Commun. **8**, 263 (1970).

[110] B. Segall, M. R. Lorenz, and R. E. Halsted, Electrical Properties of n-Type CdTe, Phys. Rev. **129**, 2471 (1963).

[111] F. T. J. Smith, Electrically active point defects in cadmium telluride, Metall. Mater. Trans. B **1**, 617 (1970).

[112] M. Shinohara, M. Yamanaka, H. Daimon, E. Sakuma, H. Okumura, S. Misawa, K. Endo, and S. Yoshida, Growth of High-Mobility 3C-SiC Epilayers by Chemical Vapor Deposition, Jpn. J. Appl. Phys. **27**, L434 (1988).

[113] F. Meng, J. Ma, J. He, and W. Li, Phonon-limited carrier mobility and temperature-dependent scattering mechanism of 3C-SiC from first principles, Phys. Rev. B **99**, 045201 (2019).

[114] R. L. Petritz and W. W. Scanlon, Mobility of Electrons

and Holes in the Polar Crystal, PbS, Phys. Rev. **97**, 1620 (1955).

[115] R. S. Allgaier and W. W. Scanlon, Mobility of Electrons and Holes in PbS, PbSe, and PbTe between Room Temperature and 4.2 k, Phys. Rev. **111**, 1029 (1958).

[116] J. Cao, J. D. Querales-Flores, A. R. Murphy, S. Fahy, and I. Savić, Dominant electron-phonon scattering mechanisms in n-type PbTe from first principles, Phys. Rev. B **98**, 205202 (2018).

[117] M. Karakus, S. A. Jensen, F. D'Angelo, D. Turchinovich, M. Bonn, and E. Cánovas, Phonon–Electron Scattering Limits Free Charge Mobility in Methylammonium Lead Iodide Perovskites, J. Phys. Chem. Lett. **6**, 4991 (2015).

[118] R. L. Milot, G. E. Eperon, H. J. Snaith, M. B. Johnston, and L. M. Herz, Temperature-Dependent Charge-Carrier Dynamics in $CH_3NH_3PbI_3$ Perovskite Thin Films, Adv. Funct. Mater. **25**, 6218 (2015).

[119] S. Poncé, M. Schlipf, and F. Giustino, Origin of Low Carrier Mobilities in Halide Perovskites, ACS Energy Lett. **4**, 456 (2019).

[120] C. G. Fonstad and R. H. Rediker, Electrical Properties of High-Quality Stannic Oxide Crystals, J. Appl. Phys. **42**, 2911 (1971).

[121] W. Albers, C. Haas, H. J. Vink, and J. D. Wasscher, Investigations on SnS, J. Appl. Phys. **32**, 2220 (1961).

[122] L.-D. Zhao, S.-H. Lo, Y. Zhang, H. Sun, G. Tan, C. Uher, C. Wolverton, V. P. Dravid, and M. G. Kanatzidis, Ultralow thermal conductivity and high thermoelectric figure of merit in SnSe crystals, Nature **508**, 373 (2014).

[123] J. Ma, Y. Chen, and W. Li, Intrinsic phonon-limited charge carrier mobilities in thermoelectric SnSe, Phys. Rev. B **97**, 205207 (2018).

[124] C. H. Champness and A. L. Kipling, The Hall and Seebeck effect in nonstoichiometric bismuth telluride, Can. J. Phys. **44**, 769 (1966).

[125] C. B. Satterthwaite and R. W. Ure, Electrical and Thermal Properties of Bi_2Te_3, Phys. Rev. **108**, 1164 (1957).

[126] G.-K. Ren, S. Wang, Z. Zhou, X. Li, J. Yang, W. Zhang, Y.-H. Lin, J. Yang, and C.-W. Nan, Complex electronic structure and compositing effect in high performance thermoelectric BiCuSeO, Nat. Commun. **10**, 2814 (2019).

[127] C. Jacoboni, C. Canali, G. Ottaviani, and A. Alberigi Quaranta, A review of some charge transport properties of silicon, Solid State Electron. **20**, 77 (1977).

[128] C. SuŁkowski, A. ChuchmaŁa, A. J. Zaleski, M. Matusiak, J. Mucha, P. GŁuchowski, and W. Stręk, Transport properties, specific heat and thermal conductivity of GaN nanocrystalline ceramic, J. Solid State Chem. **183**, 2501 (2010).

[129] S. K. Sutadhar and D. Chattopadhyay, Thermoelectric power of n-GaAs, J. Phys. C: Solid State Phys. **12**, 1693 (1979).

[130] A. Amith, I. Kudman, and E. F. Steigmeier, Electron and Phonon Scattering in GaAs at High Temperatures, Phys. Rev. **138**, A1270 (1965).

[131] I. Kudman and E. F. Steigmeier, Thermal Conductivity and Seebeck Coefficient of InP, Phys. Rev. **133**, A1665 (1964).

[132] T. Tsubota, M. Ohtaki, K. Eguchi, and H. Arai, Thermoelectric properties of Al-doped ZnO as a promising oxide material for high-temperature thermoelectric conversion, J. Mater. Chem. **7**, 85 (1997).

[133] K. Morikawa, Seebeck Effect in Cadmium Sulfide, J. Phys. Soc. Jpn. **20**, 786 (1965).

[134] H. Wang, E. Schechtel, Y. Pei, and G. J. Snyder, High Thermoelectric Efficiency of n-type PbS, Adv. Energy Mater. **3**, 488 (2013).

[135] D. F. Morgan and D. A. Wright, Electrical properties of single crystals of antimony-doped stannic oxide, Br. J. Appl. Phys. **17**, 337 (1966).

[136] T. H. Geballe and G. W. Hull, Seebeck Effect in Silicon, Phys. Rev. **98**, 940 (1955).

[137] C. Herring, Theory of the Thermoelectric Power of Semiconductors, Phys. Rev. **96**, 1163 (1954).
