# Supplementary material for: Efficient calculation of carrier scattering rates from first principles
Source: Nat Commun. 2021 Apr 13;12:2222. doi: 10.1038/s41467-021-22440-5 (PMC8044096; doi:10.1038/s41467-021-22440-5)
Supplement: Supplementary file 2 — Description of Additional Supplementary Files [file 41467_2021_22440_MOESM2_ESM.pdf]

Description of Additional Supplementary Files

Supplementary Software 1

AMSET software used to calculate all results in the main text.
