# Supplementary material for: Efficient calculation of carrier scattering rates from first principles
Source: Nat Commun. 2021 Apr 13;12:2222. doi: 10.1038/s41467-021-22440-5 (PMC8044096; doi:10.1038/s41467-021-22440-5)
Supplement: Supplementary file 3 — Supplementary Software 1 [file 41467_2021_22440_MOESM3_ESM.zip › amset-src/docs/theme/overrides/partials/footer.html]

{#-
This file was automatically generated - do not edit
-#}
{% import "partials/language.html" as lang with context %}
{% if page.previous\_page or page.next\_page %}

{% if page.previous\_page %}

{% include ".icons/material/arrow-left.svg" %}

{{ lang.t("footer.previous") }}
{{ page.previous\_page.title }}

{% endif %}
{% if page.next\_page %}

{{ lang.t("footer.next") }}
{{ page.next\_page.title }}

{% include ".icons/material/arrow-right.svg" %}

{% endif %}

{% endif %}
