# Supplementary figures and images for: Efficient calculation of carrier scattering rates from first principles
Source: Nat Commun. 2021 Apr 13;12:2222. doi: 10.1038/s41467-021-22440-5 (PMC8044096; doi:10.1038/s41467-021-22440-5)

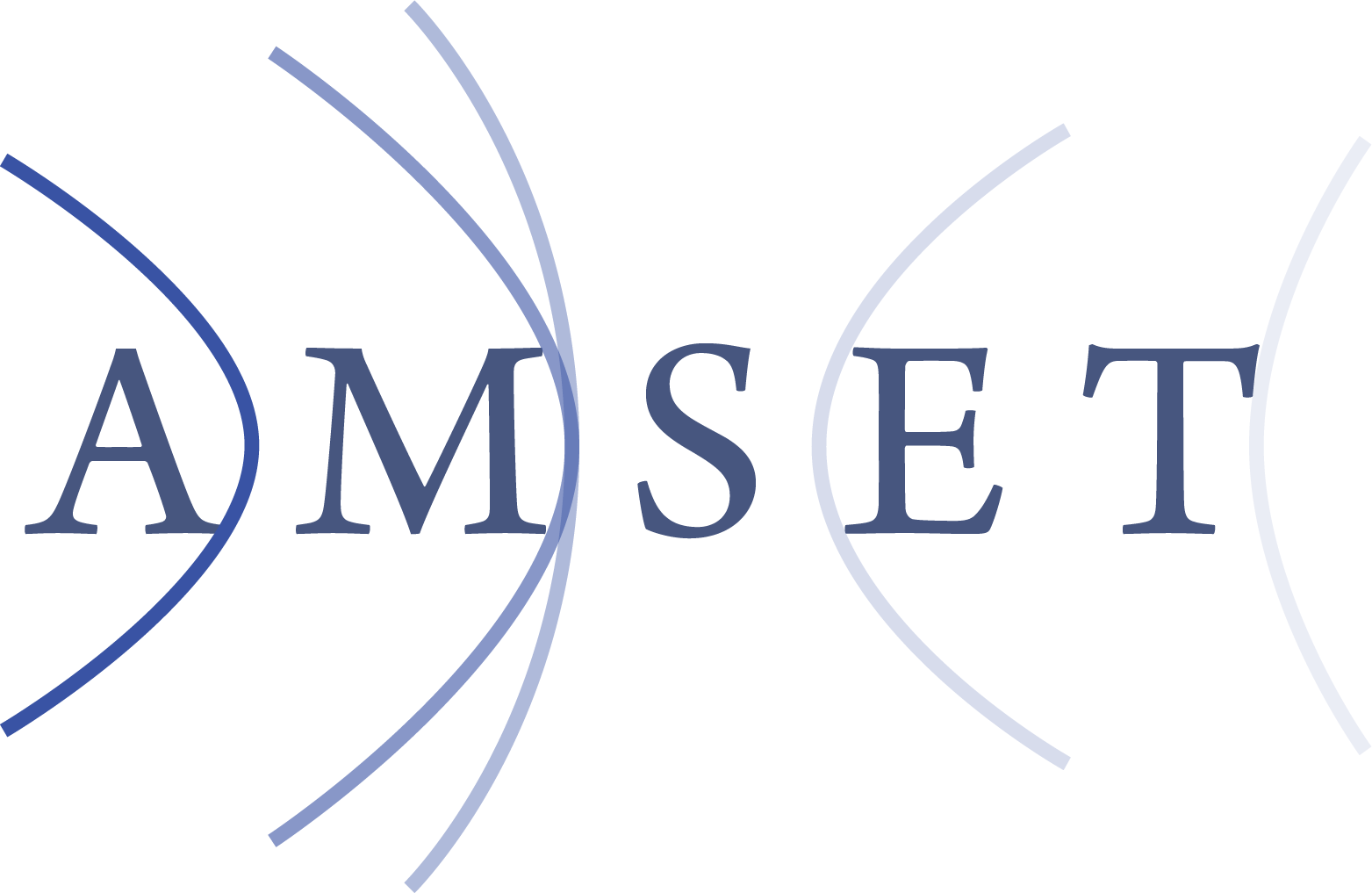

Supplement: Supplementary file 3 — Supplementary Software 1 [file 41467_2021_22440_MOESM3_ESM.zip › amset-src/docs/src/amset_logo.png]

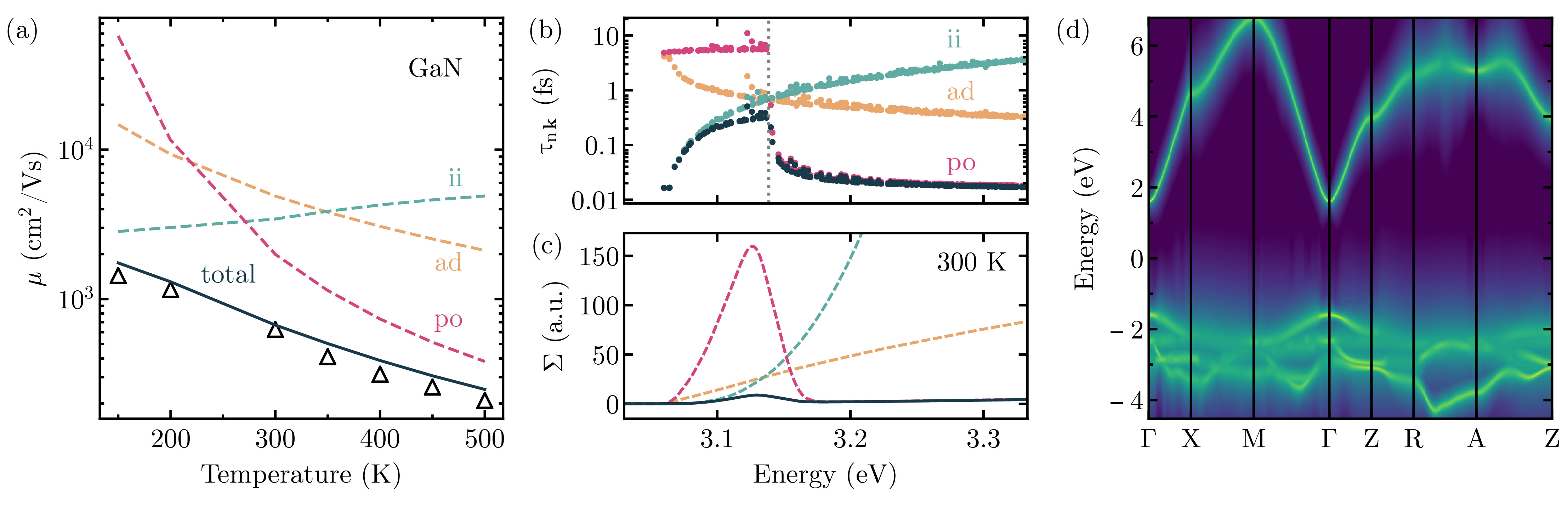

Supplement: Supplementary file 3 — Supplementary Software 1 [file 41467_2021_22440_MOESM3_ESM.zip › amset-src/docs/src/properties.jpg]
